# Genomic Signals of Local Adaptation Associated With Environmental Variables in *Eleginops maclovinus* From Northern Chilean Patagonia

**DOI:** 10.1002/ece3.71524

**Published:** 2025-06-24

**Authors:** C. Eliza Claure, Garrett J. McKinney, José Dellis Rocha, José M. Yáñez, Iván Pérez‐Santos, Cristian B. Canales‐Aguirre

**Affiliations:** ^1^ Centro i~mar Universidad de Los Lagos Puerto Montt Chile; ^2^ Núcleo Milenio INVASAL Concepción Chile; ^3^ Programa de Magister en Ciencias, mención Producción, Manejo y Conservación de Recursos Naturales Universidad de Los Lagos Puerto Montt Chile; ^4^ Programa de Doctorado en Ciencias, mención Conservación y Manejo de Recursos Naturales Universidad de Los Lagos Puerto Montt Chile; ^5^ Washington Department of Fish and Wildlife Seattle Washington USA; ^6^ Escuela de Obstetricia, Facultad de Ciencias Para el Cuidado de la Salud Universidad San Sebastián Sede de La Patagonia Puerto Montt Chile; ^7^ Facultad de Ciencias Veterinarias y Pecuarias Universidad de Chile Santiago Chile; ^8^ Center for Oceanographic Research COPAS Sur‐Austral and COPAS COASTAL Universidad de Concepción Concepción Chile; ^9^ Centro de Investigaciones en Ecosistemas de la Patagonia (CIEP) Coyhaique Chile

**Keywords:** candidate genes, Patagonian blennie, RAD‐seq, seascape genomics, SNPs

## Abstract

Understanding the evolutionary mechanisms that shape the adaptive divergence across spatially heterogeneous environments is a challenging task for evolutionary ecologists. The Chilean marine Patagonia is a complex ecosystem with diverse geomorphology and physical–chemical oceanographic conditions. There is limited research evaluating the interactions between selective forces and environmental conditions in this area. This study focuses on identifying the genomic signals of local adaptation of the endemic marine fish, *Eleginops maclovinus* , from Chilean North Patagonia. To achieve this goal, we used an environmental marine database (temperature, salinity, oxygen, phosphate, nitrate and silicate) collected from 1995 to 2018 and 11,961 SNPs obtained from 246 individuals from 10 sampling locations across this area. We identified putative adaptive loci using 10 bioinformatic software tools, where five were based on population genetic differentiation (PGD) and five based on genotype‐environment association (GEA). We identified 392 adaptive loci using PGD and 2164 associated with at least one of the six environmental variables analyzed using GEA. A total of 131 loci were shared between the PGD and GEA approaches, of which 37 were associated with genes involved in growth, metabolism, and homeostasis. Then, we evaluated the variation of adaptive loci with environmental variables using polygenic scores and found significant correlations with temperature, salinity, and oxygen, indicating polygenic selection along environmental gradients. This study highlights how polygenic selection drives local adaptation in *Eleginops maclovinus* and underscores the value of integrating genomic and environmental data for conservation in the Patagonian ecosystem.

## Graphical abstract

Studying evolutionary processes in the unique and understudied Patagonian fjord ecosystem is a challenging task for evolutionary ecologists. Genomic signals of local adaptation are present in a species from northern Chilean Patagonia. Polygenic selection drives local adaptation in *Eleginops maclovinus* .

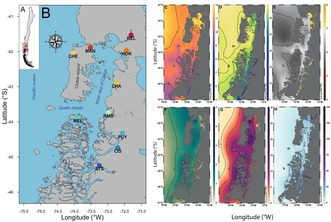

## 1 Introduction

The main aim of adaptive genomics is understanding the molecular basis of local adaptation in species inhabiting spatially heterogeneous environments. Environmental factors such as temperature, salinity, and nutrient concentration play a crucial role in the occurrence of local adaptations in marine populations. While many fish species exhibit characteristics such as high fecundity, large population sizes, pelagic larval phases, and extensive geographical distributions (Palumbi 2003; Pascual et al. 2017), which typically favor high gene flow and could hinder local adaptation. Recent genomic studies provided robust evidence of adaptation associated with various environmental variables in different fish species (Bekkevold et al. 2020; Andrews et al. 2023; Fuentes‐Pardo et al. 2024; Khrustaleva 2024). These findings underscore the importance of identifying genetic markers to understand adaptation in marine ecosystems.

Identifying genomic signals associated with local adaptation is essential for preserving the genetic diversity of species, particularly in the context of climate change and anthropogenic pressures (Hoban et al. 2020; Laikre et al. 2020). Given the rising temperatures and salinity levels in ecosystems such as the Patagonian fjords (Iriarte et al. 2010; Aguayo et al. 2019; Marquet et al. 2023), predicting adaptive responses is vital for the conservation of species in these environments. Key biological traits in fish, including embryonic survival, larval metabolism, and developmental rates, are strongly influenced by environmental variables such as temperature, salinity, and oxygen levels (Nikinmaa and Rees 2005; Gagliano et al. 2007; Dowling and Simmons 2009; Lehtonen and Kvarnemo 2015; Hossain Aunkor et al. 2025). Therefore, examining both neutral and adaptive genetic variation through high‐throughput sequencing is essential to identify appropriate management units and ensure the conservation of the species' genetic diversity.

Restriction site‐associated DNA sequencing (RAD‐seq) leverages high‐throughput sequencing to genotype individuals by using restriction enzymes to reduce genome complexity (Miller et al. 2007; Baird et al. 2008; Andrews et al. 2016; McKinney, Larson, et al. 2017). The use of hundreds of thousands of RAD‐seq markers for exhibiting the signatures of selection is increasingly applied to marine organisms, offering particular advantages for complex genomes in the absence of a reference sequence (Li et al. 2019; Segovia et al. 2020; O'Leary et al. 2021; Fuentes‐Pardo et al. 2024). For identifying adaptive loci, there are two predominant genome scan approaches: (i) population genetic differentiation (PGD) and (ii) genotype‐environment association (GEA). Distinguishing genetic signals from adaptive and neutral processes relies on differing algorithms, models, and assumptions, often leading to false positives (de Villemereuil et al. 2014; Rellstab et al. 2015; de Villemereuil and Gaggiotti 2015; François et al. 2016). Therefore, combining PGD and GEA methods offers a more efficient strategy to detect local adaptation while minimizing such errors (de Villemereuil et al. 2014; Gagnaire et al. 2015; François et al. 2016).

Northern Chilean Patagonia encompasses approximately 140,000 km^2^, extending from ~41.5° S (Reloncaví Fjord) to ~46.5° S (San Rafael Lagoon) (Rodrigo 2008). Its ecosystems are composed of an extensive and interconnected system of fjords, channels, gulfs, estuaries, and bays. These coastal environments are influenced by complex physical regimes—including freshwater input from rivers and ice fields, strong vertical stratification, and estuarine circulation—which can significantly modulate biological productivity by affecting nutrient availability, light penetration, and phytoplankton dynamics (Pantoja et al. 2011; Iriarte et al. 2014). Oceanographically, Northern Chilean Patagonia is considered a transitional marine system, where the interaction between marine and fresh waters produces strong vertical and horizontal gradients in physical–chemical variables (Iriarte et al. 2014; Pérez‐Santos et al. 2014; Linford et al. 2024). These characteristics make Patagonia an excellent natural laboratory to investigate how seascape heterogeneity shapes genomic patterns. Integrating genomic and environmental data provides a powerful framework for uncovering the mechanisms driving adaptive divergence in ecologically complex and connected marine ecosystems. However, in Northern Patagonia, seascape genomics remains an emerging field, with only a handful of studies addressing the genomic basis of local adaptation or its association with environmental gradients (Araneda et al. 2016; Canales‐Aguirre et al. 2016, 2018, 2022; Delgado et al. 2019; Segovia et al. 2020). Key knowledge gaps remain regarding the spatial scale and consistency of adaptive signals across taxa and habitats.

*Eleginops maclovinus* (Cuvier and Valenciennes 1830) is a notothenioid fish (Perciformes) of the monotypic family Eleginopidae (Pequeño 1989). This marine fish is endemic to the coastal temperate and sub‐Antarctic waters of South America with a distribution range along the Atlantic and Pacific Patagonian coasts. In the Atlantic Ocean, it is distributed from Uruguay (~35° S) to the Beagle Channel (~54° S), including the Falkland/Malvinas Islands (Gosztonyi and Ae 1974; Eastman 1993), while in the Pacific Ocean, it ranges from the Beagle Channel to Valparaíso (~33° S) (Pequeño 1989). This species is a protandrous hermaphrodite with the highest fecundity (~796 eggs/g) (Brickle et al. 2005) among notothenioids (80–300 eggs/g) (Kock 1992; North 2001). It is euryhaline and can tolerate a broad range of salinity concentrations (5 and 45 PSU); however, extreme salinities can produce dramatic metabolic responses to fuel osmoregulatory tissues (Vargas‐Chacoff et al. 2014; Vargas‐Chacoff, Moneva, et al. 2015). Furthermore, juveniles of *E. maclovinus* have been described as having a large eurythermal capacity, with tolerance between 4°C and 20°C (Vanella et al. 2012; Oyarzún et al. 2018; Lattuca et al. 2018). All these studies described above have been conducted under controlled conditions; they focus on one or two environmental variables and do not associate with genetic data. The latter leaves a gap in how loci under selection can shape the adaptive capacity of *E. maclovinus* populations, considering the complex environmental variability in estuaries and fjords.

Studies about the geographic pattern of genetic diversity in *E. maclovinus* are limited to three (Ceballos et al. 2012, 2016; Canales‐Aguirre et al. 2022). A phylogeographic research conducted by Ceballos et al. (2012) using mitochondrial DNA sequences for nine locations did not find any evidence for the structuring of genetic variation into an Atlantic and a Pacific group. Subsequently, using nine microsatellites revealed a low but significant level of genetic differentiation between Pacific and Atlantic populations and differences between locations in the Pacific coast (Ceballos et al. 2016). However, a single mitochondrial locus or nine microsatellites may not be robust enough to allow them to recover significant subdivision among geographic regions. Recently, Canales‐Aguirre et al. (2022) used 12,050 SNPs in five locations distributed across Northern Patagonia to identify neutral and adaptive population structure. However, that study included only five locations and used the Bio‐ORACLE environmental database (Tyberghein et al. 2012; Assis et al. 2018) which lacks fine‐scale resolution for fjord areas like Patagonia. In addition, its focus was to use only three approaches to identify neutral and adaptive loci for conservation and management purposes.

In this study, we applied RAD‐seq sequencing to generate SNPs genotyped in 246 individuals of *E. maclovinus* collected in North Patagonia. Here, we expanded the sampling locations from Canales‐Aguirre et al. (2022), increasing the number to 10 locations to better capture the environmental heterogeneity of their North Patagonian seascape. Our aim was to detect SNPs putatively under selection using population genetic differentiation (PGD) and genotype‐environment association (GEA) approaches. We tested associations between SNPs and six environmental variables (temperature, salinity, oxygen, nitrate, phosphate and silicate) obtained from oceanographic data recorded between 1995 and 2018 in this area. We implemented a BLASTx search on putative adaptive SNPs identified by both PGD and GEA approaches to investigate genes with molecular functions potentially involved in local adaptation. Then, we evaluated the variation of adaptive loci using polygenic scores where we found significant correlations and supporting polygenic selection in North Patagonia.

## 2 Materials and Methods

### 2.1 Study Area and Environmental Predictors

**FIGURE 1:**
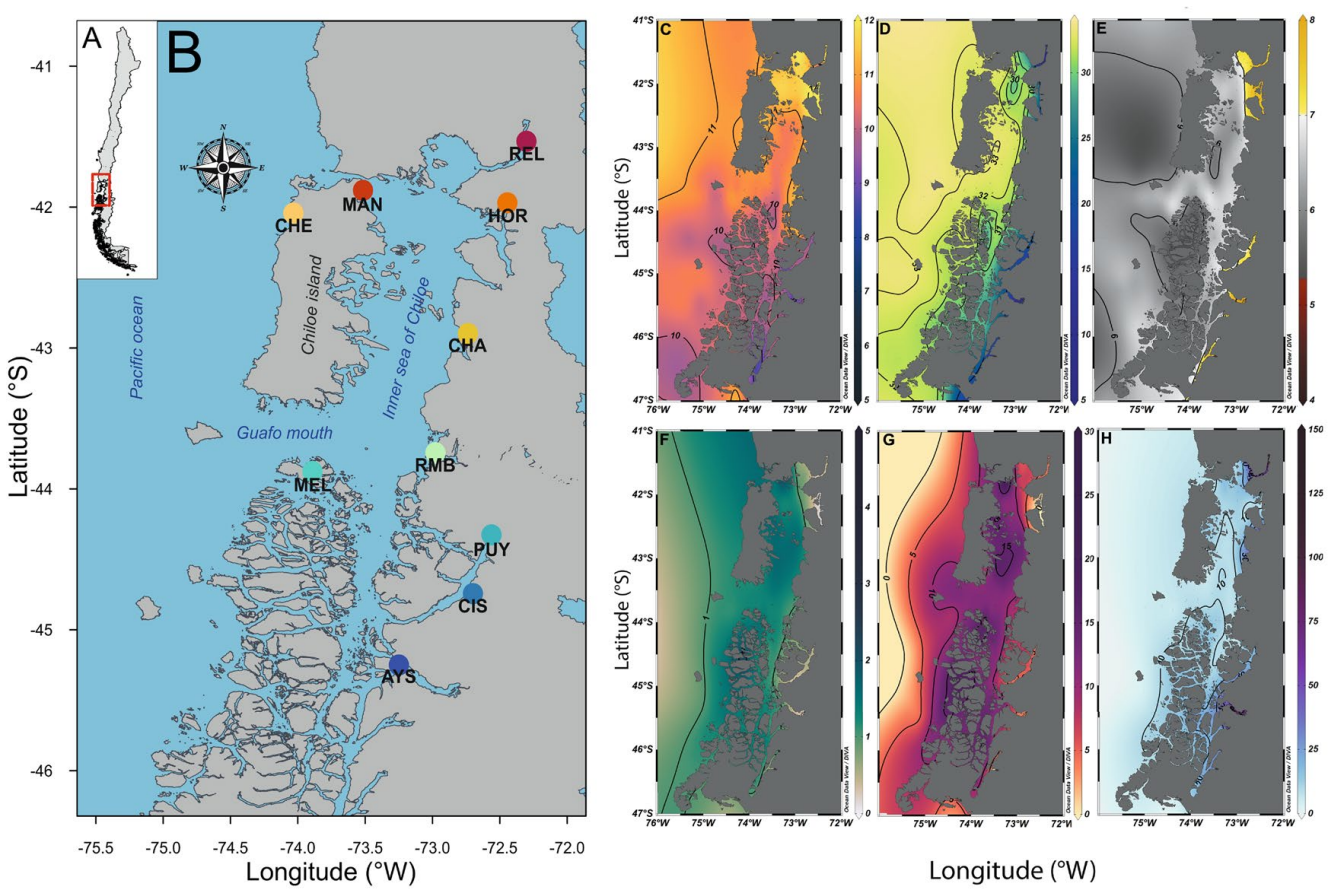
Geographic area and environmental variables used. (A) Map of sampling locations in South America. (B) Sampling site locations on North Patagonian fjord ecosystems in southern Chile REL: Reloncaví Estuary (*n* = 17), MAN: Manao (*n* = 23), HOR: Hornopirén (*n* = 24), CHE: Chepu (*n* = 12), CHA: Chaitén (*n* = 15), RMB: Raúl Marín Balmaceda (*n* = 22), MEL: Melinka Island (*n* = 20), PUY: Puyuhuapi (*n* = 22), CIS: Port Cisnes (*n* = 20), AYS: Port Aysén (*n* = 27). (C) mean surface temperature (°C); (D) mean surface salinity (g/kg); (E) mean surface concentration oxygen (mL/L); (F) mean surface nitrate (μM); (G) mean surface phosphate (μM); (H) mean surface silicate (μM).

Chilean Patagonia has been affected by ice sheets calving into the ocean, retraction of the sea coastline, and a decrease in marine water temperatures since the Great Patagonian Glaciation (c. 1 Ma) during the Pleistocene (Rabassa et al. 2011). The Northern Chilean Patagonia (Figure 1A) is characterized by interactions between marine and freshwater, generating sharp vertical and horizontal salinity gradients (Dávila et al. 2002; Pérez‐Santos et al. 2014; Schneider et al. 2014). The temperature shows a latitudinal gradient, which decreases southward until the Antarctic Polar Front (~55° S) (Iriarte et al. 2014). The oxygen concentration in the fjords is the result of horizontal advection of adjacent well‐oxygenated Subantarctic Waters (5–6 mL/L) representing the major source of oxygen in the deep layers of the inner seas of Patagonia (Silva and Vargas 2014). In recent years, dissolved oxygen in the fjords of Chilean Patagonia has decreased by 1%–2%, potentially driven by nutrient accumulation and rising temperatures (Linford et al. 2023a, 2023b, 2024). The concentrations of inorganic nutrients show a strong seasonal signal, with high nitrate and orthophosphate concentrations during winter and lower values during spring, presumably caused by a severe increase in primary productivity when light availability in near‐surface waters increases (Iriarte et al. 2007; González et al. 2011; Torres et al. 2014; Jacob et al. 2014). The concentration of silicic acid shows a latitudinal and longitudinal gradient due to surface water mixing between nitrate‐rich but silicic acid‐depleted oceanic subantarctic waters and silicic acid‐rich but nitrate‐depleted continental waters (Aracena et al. 2011; González et al. 2011; Torres et al. 2014).

We compiled an environmental database using data collected through the CIMAR Program of the National Oceanographic Committee of Chile (CONA), from CIMAR 1 (1995) to CIMAR 24 (2018). The database comprises 1195 sampling stations located along the inner sea of Chiloé Island, estuaries, channels, and fjords from the Chilean Patagonia sampled in spring and winter (http://www.shoa.cl/n_cendhoc/productos/reporte_datos.php) (Figure S1). At each sampling station, environmental variables were measured from the surface to the maximum possible depth. Considering that traditional databases lack high‐resolution data near the coasts (e.g., Bio‐Oracle, World Ocean Atlas, Aqua/MODIS), we highlight the use of information obtained by CIMAR as an effective alternative to detect the environmental heterogeneity of Northern Patagonia. Our database compiled contains six environmental predictors: conservative temperature (°C), absolute salinity (g/kg), oxygen (mL/L), nitrate (μM), phosphate (μM) and silicate (μM) concentrations (Figure 1C–H). We used Ocean Data View v.5.4.0 (Schlitzer 2002) for data visualization. We estimated the mean, minimum, maximum, and range of these environmental variables at each of the 10 locations within the upper 100 m of the water column, using data from the full period by the database (1995–2018) (Table S1). This is particularly relevant for *E. maclovinus* , which inhabits coastal and estuarine areas at depths < 40 m, with spawning occurring during the spring (Brickle et al. 2005).

### 2.2 Sampling and Genotyping

Between November 2018 and April 2019, we sampled 246 individuals of *E. maclovinus* from 10 locations (sample size varied from 12 to 27) throughout the North Patagonian fjord ecosystems in southern Chile (Figure 1B and Table S2). Five of the 10 sampling locations included in this study were previously analyzed by Canales‐Aguirre et al. (2022). Sampling, DNA extraction, RAD‐sequencing library preparation, sequencing with Illumina technology, and the pipeline of bioinformatic analyses were performed following procedures described by Canales‐Aguirre et al. (2022).

Loci and samples were filtered iteratively by initially removing poor‐quality data, recalculating missing data proportions, and applying increasingly stringent thresholds, following McKinney et al. (2020). The first part of the filtration process consisted of excluding SNPs or individuals based on the following criteria: (i) minor allele frequencies (MAF) per sampling location, (ii) genotype call rate per locus, (iii) genotype call rate per sample. Initially, SNPs were filtered using a MAF threshold of ≤ 0.05 and a genotype call rate threshold of 50% per locus and per sample. In a subsequent filtering step, a stricter MAF threshold of ≤ 0.1 and a genotype call rate threshold of 90% per locus and per sample were applied. Due to gene duplication events described to have affected the evolution of the notothenioid genome (Xu et al. 2008; Chen et al. 2008, 2019), *HDplot* (McKinney, Waples, et al. 2017) was used to identify paralogs, which were removed from further analysis (parameter flags: *H* < 0.6; |*D*| < 5). We removed SNPs in Hardy–Weinberg disequilibrium (*p*‐value < 0.05) if they deviated in three or more locations to avoid potential issues such as inbreeding, population stratification, and genotyping errors in the identified SNPs (Wigginton et al. 2005). For reads with more than one SNP, we only kept those with the highest *F*_ST_ to reduce the influence from linked loci in the results. The resulting filtered (.vcf) file was converted into the corresponding file formats for the subsequent analyses by using *PGDSpider* v. 2.1.1.5 (Lischer and Excoffier 2012).

### 2.3 Detecting Putative Loci Under Selection

We employed 10 different softwares to identify putative adaptive loci: five based on population genetic differentiation (PGD) and five on genotype‐environment association (GEA). The PGD approach detects loci under selection by comparing the genetic differentiation index (*F*_ST_) and other metrics (e.g., *F*_IS_, homozygosity excess) against a neutral evolution model. Higher *F*_ST_ values at specific loci indicate selection favoring certain alleles (Beaumont 2005; François et al. 2016). Contrarily, GEA searches for associations between environmental variables and allele frequencies across a set of populations spread over a geographic space (Rellstab et al. 2015; Hoban et al. 2016). We combined multiple approaches to minimize false positive rates and maximize our chances of detecting genomic signals of local adaptation (Rellstab et al. 2015; François et al. 2016). For the PGD approach, we used *fsthet* v.1.0.1 (Flanagan and Jones 2017), *BayeScan* v.2.1 (Foll and Gaggiotti 2008), *OutFLANK* v.0.2 (Whitlock and Lotterhos 2015), *PCAdapt* v.4.3.3 (Luu et al. 2017; Privé et al. 2020), and *Arlequin* v.3.5.2.2 (Excoffier and Lischer 2010) (Table S3). For the GEA approach, we used six environmental variables and the following softwares: *Latent Factors Mixed Model* (*LFMM*) (Frichot et al. 2013), *BayeScEnv* (de Villemereuil and Gaggiotti 2015), *Redundancy Analysis* (*RDA*) (Hair et al. 1995; Zuur et al. 2010; Capblancq et al. 2018; Capblancq and Forester 2021), *Moran Spectral Outlier Detection* (*MSOD*) (Wagner et al. 2017), and *Samβada* (Stucki et al. 2017; Duruz et al. 2019) (Table S4). In all programs, a 5% False Discovery Rate and respective *p*‐values or *q*‐values were applied to detect putative adaptive loci. We employed *UpSet* diagrams (Lex et al. 2014) to visually compare the results from the 10 software programs, identifying loci flagged as outliers by at least two programs as putative adaptive loci. For subsequent analyses, loci were classified into two categories: putative adaptive loci (under directional selection) and putative neutral loci, excluding putative loci under balancing selection.

### 2.4 Genetic Diversity and Population Structure

For the adaptive and neutral datasets, we determined, described, and compared overall and population‐specific genetic diversity. The expected heterozygosity (*H*_E_), observed heterozygosity (*H*_O_) and inbreeding coefficient (*F*_IS_) were calculated for each locus and location using the *Hierfstat* v 0.04.10 R package (Goudet 2005). We assessed the genetic differentiation with pairwise *F*_ST_ (Weir and Cockerham 1984) comparisons between locations using the R package *StAMPP* v.1.6.2 (Pembleton et al. 2013) and the statistical significance (*p* < 0.05) was determined using 10,000 permutations.

We used two approaches to investigate the genetic structure: Bayesian clustering program *Structure* v.2.3.4. (Pritchard et al. 2000) and discriminant analysis of principal components (DAPC) (Jombart et al. 2010) using the R package *adegenet* v.2.1.5. (Jombart 2008; Jombart and Ahmed 2011). *Structure* is a Bayesian method that uses a Markov chain Monte Carlo (MCMC) algorithm based on the Gibbs sampler algorithm to identify the number of putative genetic clusters. This method assumes Hardy–Weinberg equilibrium (HWE) and linkage equilibrium among loci in sample population individuals (Pritchard et al. 2000). We conducted the analysis using 10 replicates per *K* value for *K* = 1 to *K* = 10, with 10,000 burn‐in steps and 100,000 MCMC‐sampling steps. The runs were collected, sorted, and merged and functioned in the R package *pophelper* (Francis 2017) to produce assignment barplots. The most likely number of genetic clusters (*K*) was chosen using the Δ*K* method (Evanno et al. 2005). The DAPC method identifies and describes clusters of genetically related individuals from genomic datasets and allows the optimal visualization of between‐population differentiation in multivariate space (Jombart et al. 2010). First, we carried out a principal component analysis (PCA) and used the principal components (PCs) thus produced as synthetic variables for a discriminant analysis, as outlined in Jombart et al. (2010). The optimal number of principal components was determined by cross‐validation (*xvalDapc* function (Jombart et al. 2010)) to avoid overfitting.

We also conducted isolation by distance (IBD) analyses to test whether genetic distance increased with geographic distance with adaptive and neutral data. We assessed the correlation between the linearized pairwise *F*_ST_ matrix and the Euclidean (geographic) distance matrix using a Mantel test as implemented in the R package *vegan* (Oksanen et al. 2016). Statistical significance was assessed with 999 permutations. To test whether the correlation between genetic and geographic distances is a result of a continuous or distant patchy cline of genetic differentiation, local densities of distances to disentangle both processes were plotted using two‐dimensional kernel density estimation (*kde2d* function) of the *MASS* R package (Venables and Ripley 2013).

### 2.5 Additive Polygenic Scores (APS)

We assessed cumulative adaptive genetic variation by calculating additive polygenic scores (APS) associated with each environmental variable (Gagnaire and Gaggiotti 2016; Babin et al. 2017). For this analysis, we prioritized three biologically important environmental variables: annual maximum temperature (°C), annual maximum salinity (g/kg), and annual minimum oxygen concentration (mL/L) (Holliday 1969; Nikinmaa and Rees 2005; Brian et al. 2008; Vanella et al. 2012; Vargas‐Chacoff et al. 2014). We prioritized the maximum values of temperature and salinity considering the effect that climate change has on them. According to the Community Climate System Model 3.0 (CCSM3) of the National Center for Atmospheric Research (NCAR), a 2.5°C increase in the temperature of Chilean coastal areas is predicted by 2055 (Silva et al. 2016). Similarly, the Center for Oceanographic Research in the Eastern South Pacific (COPAS) detected an increase of 0.25 in salinity in the upper layers of the central‐southern coast of Chile (Schneider et al. 2017). We also considered the minimum oxygen concentration, as hypoxic conditions negatively impact fish fitness (Richards 2009; Wu 2009). All these variables have an impact on the adaptive genetic diversity of several species (Pauls et al. 2013; Bernatchez 2016).

We assessed the relationship between each of the 131 putative adaptive loci (see results Section 3.2) and each environmental variable. We used genotypes (0,1,2) to determine the score for each favored allele; if the slope is positive, and (2,1,0) if negative (Babin et al. 2017). We calculated APS by summing the scores of favored alleles associated with each environmental variable. Finally, we evaluated the relationship between individual APS and environmental variables using three models: linear, quadratic, and null, determining the best fit based on the lowest Akaike information criterion (AIC) value.

### 2.6 Loci Annotation and Candidate Gene Identification

Loci identified as putative adaptive loci (see results Section 3.5) were functionally annotated through Blast2GO v.6.0.3 (Conesa and Götz 2008). We compared the sequences against bony fishes (taxa: 7898, Actinopterygii) to determine whether any putative adaptive loci were associated with genes that may be related to environmental tolerances (e.g., heat shock, immune system proteins, osmoregulation). We applied the BLASTx algorithm with a cut‐off of *E*‐value ≤ 1 × 10^−5^, homology of sequences of more than 75%, and other parameters were used as default. Subsequent analysis only considered sequences with functional annotation (blasted, mapped and annotated).

## 3 Results

### 3.1 RAD Sequencing and Data Filtering

After quality control, 2,359,848 (511,190 RADtag) putative loci were obtained for Stacks pipeline. Using the iterative filtering process (MAF and missing data), we identified a total of 2,328,560 (98.67%) putative singletons and 386 putative paralog loci; the latter were removed (1.24%). Further filtering by HWE discarded 13,215 loci (42.96%) and one SNP per tag (the highest *F*_ST_) discarded 5582 SNPs (31.82%). A total of 11,961 SNPs (5.01%) were retained from 202 individuals across the 10 sampling locations (Table 1).

**TABLE 1:** Description of steps used to filter SNPs.

Filtering steps	Sample size	Number of loci
SNP after STACKS	246	2,359,848
MAF > = 0.05^a^	246	1,494,046
Genotyped by locus (50%)^a^	246	167,752
Genotyped by sample (50%)^a^	236	167,752
Genotyped by locus (90%)^a^	236	54,694
Genotyped by sample (90%)^a^	202	54,694
MAF > = 0.1^a^	202	31,144
Singletons (HDPlot *H* = 0.6; *D* = 5)	202	30,758
Hardy–Weinberg equilibrium in 100% locations	202	17,543
one SNP by tag	202	11,961

^a^ Steps included in the iterative filtering process.

### 3.2 Identification of Loci Putatively Under Selection

**FIGURE 2:**
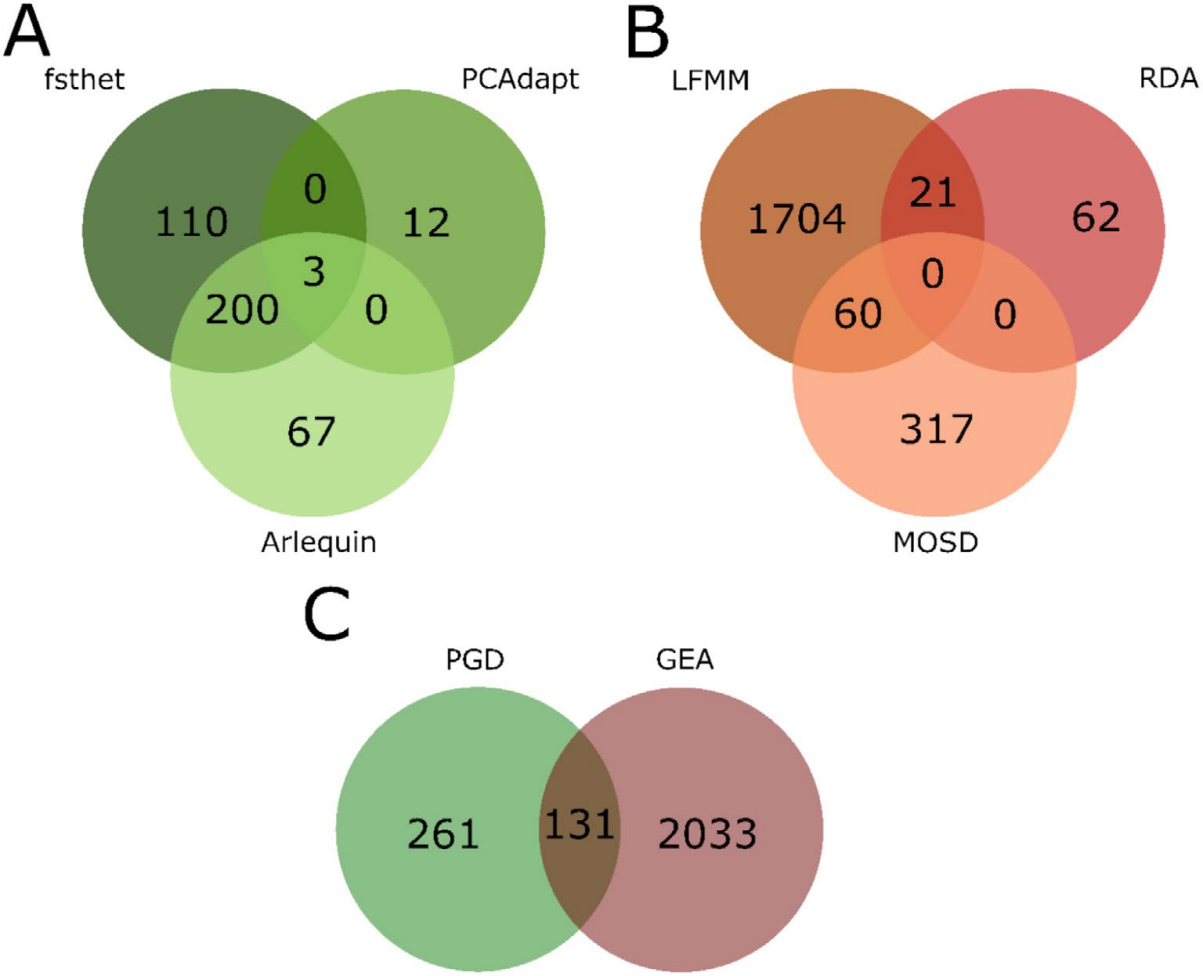
Venn diagram for all intersections of software used. (A) Loci detected by software based on population genetic differentiation (PGD), (B) loci detected by software based on environment genotype‐association, and (C) loci shared between PGD and GEA. The *OutFLANK*, *BayeScan*, *BayeScEnv*, and *Samβada* software are excluded since they did not identify any adaptive loci.

From population genetic differentiation (PGD) and genotype‐environment association (GEA) we identified different putative adaptive loci. Out of the 11,961 loci, we identified 392 loci under positive selection by PGD and 2164 loci using the GEA approach. A total of 131 loci were identified as shared between the PGD and GEA approaches (Figure 2).

**FIGURE 3:**
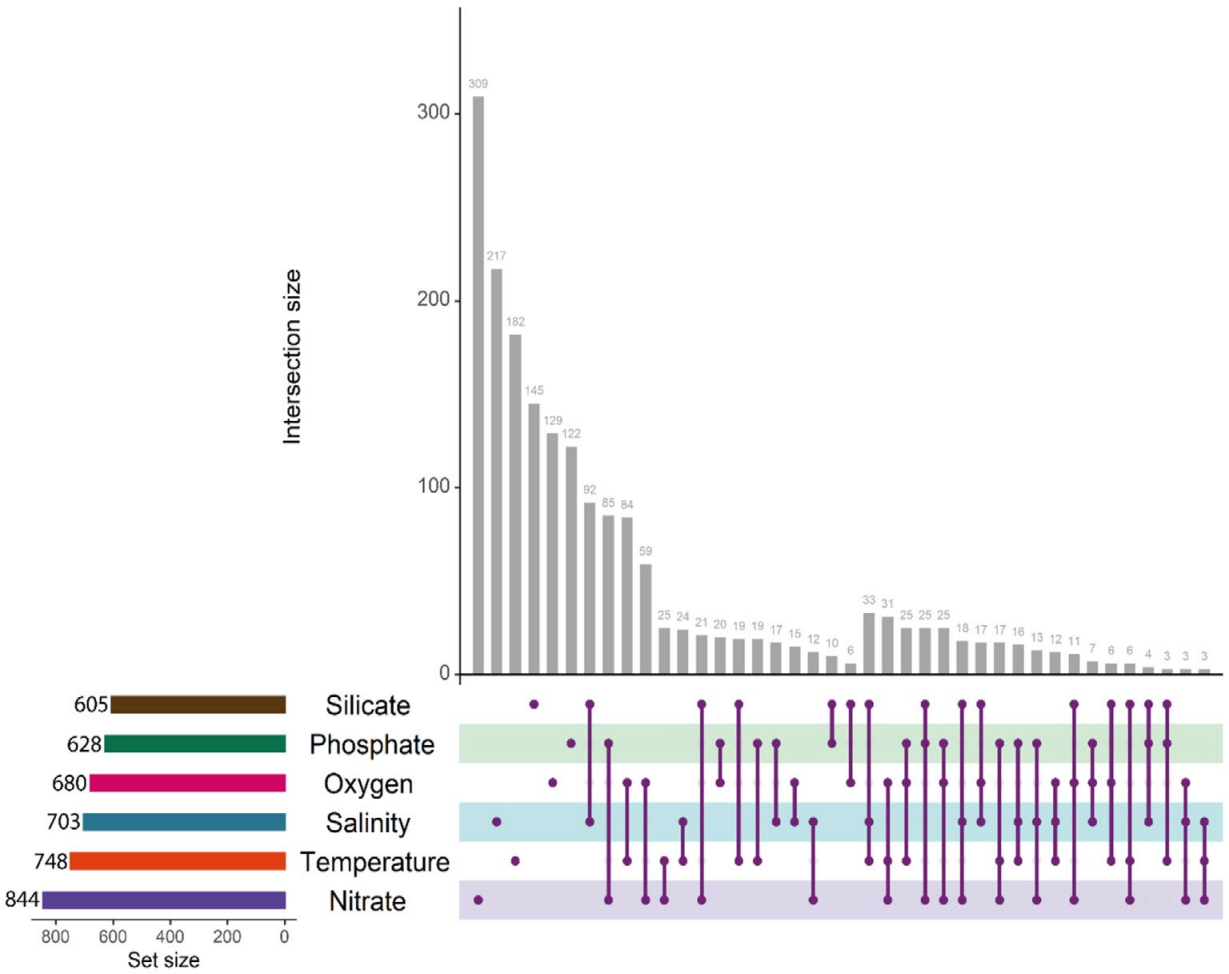
UpSet diagram: Matrix layout for all intersections of environmental variables. The set size (horizontal bars) indicates the total number of loci correlated with that environmental variable. The intersection size (vertical bars) indicates the number of loci per set intersection, the purple dots represent the set of unique loci correlated only with that environmental variable and no other one, while the purple dots connected by a line represent loci shared by two, three, or more variables.

Within PGD approaches, *fsthet* detected 334 SNPs (2.79%) under balancing selection and 313 SNPs (2.62%) under divergent selection (Figure S2). *Arlequin* identified 270 SNPs under divergent selection (2.26%) (Figure S3). *PCAdapt* detected 15 SNPs (0.13%) under divergent selection (Figure S4). *BayeScan* and *OutFLANK* found no outlier. For GEA approaches, *LFMM* identified 1785 loci under divergent selection, with the highest number of associations to nitrate concentration (*n* = 684 loci) (Figure S5). *MSOD* detected 377 loci, with the highest number of associations to temperature (*n* = 377 loci) (Figure S6). Finally, *RDA* detected 83 loci under divergent selection, with the highest number of associations to silicate (33 loci) (Figure S7). *Samβada* and *BayeScEnv* did not detect any correlation with environmental variables. Overall, across GEA approaches, the nitrate concentration (*n* = 844), temperature (*n* = 748), and salinity (*n* = 703) were the environmental variables with which most of the loci were correlated (Figure 3). Of the total of 2164 that correlated with each of the six environmental variables, 783 of the loci were significantly associated with more than one environmental variable.

### 3.3 Assessment of Genetic Diversity and Population Structure

Summary statistics of genetic diversity revealed similar values for each location within each dataset (Figure S8). Most locations showed lower values of observed heterozygosity (*H*_O_) than expected heterozygosity (*H*_E_), and positive values of inbreeding coefficient *F*_IS_. For the adaptive loci dataset, *H*_O_ 0.3364 ± 0.0078 (95% CI), *H*_E_ 0.3551 ± 0.0068 (95% CI), and *F*_IS_ 0.0528 ± 0.0120 (95% CI). For the neutral dataset, *H*_O_ 0.3414 ± 0.0074 (95% CI), *H*_E_ 0.3485 ± 0.0063 (95% CI), and *F*_IS_ 0.0206 ± 0.0117 (95% CI). Pairwise‐*F*_ST_ comparisons showed higher levels of genetic differentiation for adaptive loci (global *F*_ST_ = 0.0419 ± 0.0042 95% CI) than neutral loci (global *F*_ST_ = 0.0004 ± 0.0004 95% CI). For adaptive loci, all pairwise differentiation values were highly significant (*p*‐value ≤ 0.0004). While in neutral loci, 26 of 45 pairwise differentiation values showed significant *p*‐values (Table 2).

**TABLE 2:** Pairwise *F*_ST_ (below diagonal) and *p* value (above diagonal) for adaptive and neutral loci from North Patagonian fjord ecosystems in southern Chile.

	REL	MAN	HOR	CHE	CHA	RMB	MEL	PUY	CIS	AYS
Adaptative										
REL	—	0	0	0	0	0	0	0	0	0
MAN	0.067	—	0.0004	0	0	0	0	0	0	0
HOR	0.049	0.012	—	0	0	0	0	0	0.0004	0
CHE	0.065	0.044	0.049	—	0	0	0	0	0	0
CHA	0.066	0.050	0.050	0.053	—	0	0	0	0	0
RMB	0.044	0.030	0.023	0.050	0.050	—	0	0	0	0
MEL	0.075	0.034	0.030	0.052	0.054	0.031	—	0	0	0
PUY	0.046	0.040	0.027	0.055	0.041	0.029	0.033	—	0	0
CIS	0.059	0.034	0.021	0.055	0.043	0.025	0.035	0.023	—	0
AYS	0.047	0.044	0.020	0.055	0.046	0.027	0.043	0.026	0.029	—
Neutral										
REL	—	0	0	1	0.784	0.154	0	0.01	0.076	0.012
MAN	0.002	—	0.0004	0.991	0.325	0	0	0	0.0008	0
HOR	0.002	0.001	—	0.998	0.968	0.002	0.002	0	0.0174	0
CHE	−0.002	−0.001	−0.001	—	1	1	0.981	0.989	0.999	0.989
CHA	−0.039	0	−0.079	−0.004	—	0.852	0.876	0.256	0.931	0.481
RMB	0	0.002	0.001	−0.002	−0.047	—	0.0003	0.049	0.338	0
MEL	0.002	0.002	0.001	−0.001	−0.524	0.001	—	0	0.007	0
PUY	0.001	0.002	0.002	−0.001	0.03	0.001	0.002	—	0.0004	0.0002
CIS	0.001	0.001	0.001	−0.002	−0.068	0	0.001	0.001	—	0.0006
AYS	0.001	0.002	0.002	−0.001	0.242	0.001	0.002	0.001	0.001	—

**FIGURE 4:**
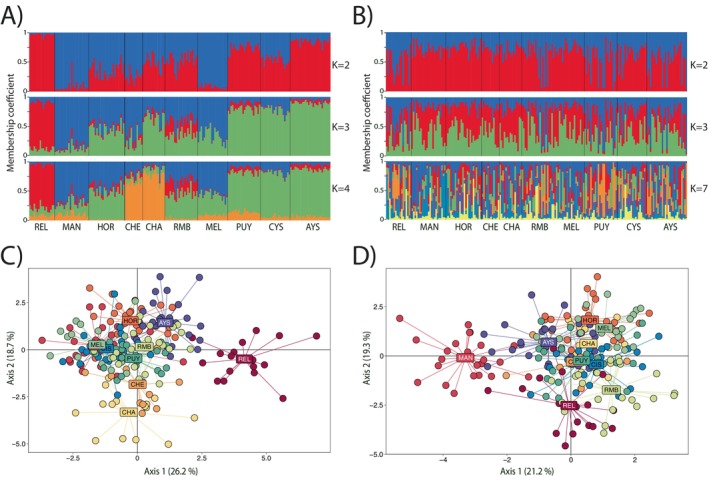
Clustering and discriminant analysis of *E*. *maclovinus* using adaptive (A, C) and neutral (B, D) loci. For clustering, each vertical line represents an individual, and colors indicate the proportion of inferred ancestry from *K* ancestral populations (*K* = 2, 3, 4 for adaptive loci; *K* = 2, 3, 7 for neutral loci). Black lines separate sampling locations. In the DPCA plots, axes correspond to the first two discriminant functions, and dots represent individuals color‐coded by sampling location.

Evanno's methods showed that the likely number of *K* clusters was *K* = 2 for adaptive loci (Figure S9) and *K* = 7 for neutral loci (Figure S10). For adaptive loci, a visual inspection revealed a clear separation of individuals for REL, MAN, and MEL using *K* = 2. We observed an additional cluster which appeared to correspond to individuals from CHE and CHA using *K* = 4 (Figure 4A). For neutral loci, *Structure* showed no clear geographical genetic structure (Figure 4B). Results from DAPC analyses revealed similar patterns to *Structure* (Figure 4C). The adaptive loci dataset showed that REL, CHA, and CHE were separate from all other locations. The first two axes explain 44.9% of the total variation. For the neutral dataset, PET, CHA, and CHE overlapped greatly with the other locations, and much less structure overall was present, although MAN seemed to be less like the other nine locations. The first two axes explain 40.5% of the total variation (Figure 4D).

The isolation by distance (IBD) analysis for adaptive loci revealed a moderate significant isolation by distance pattern (Mantel test: *r* = 0.3458, *p*‐value = 0.0320, Figure S11A), where the scatterplot of local densities of distances showed only one consistent cloud of points, indicating a continuous cline of genetic differentiation. For neutral loci, we found no detectable relationship between geographic distance and genetic distance (Mantel test: *r* = −0.0435, *p*‐value = 0.576, Figure S11B).

### 3.4 Additive Polygenic Scores (APS)

**FIGURE 5:**
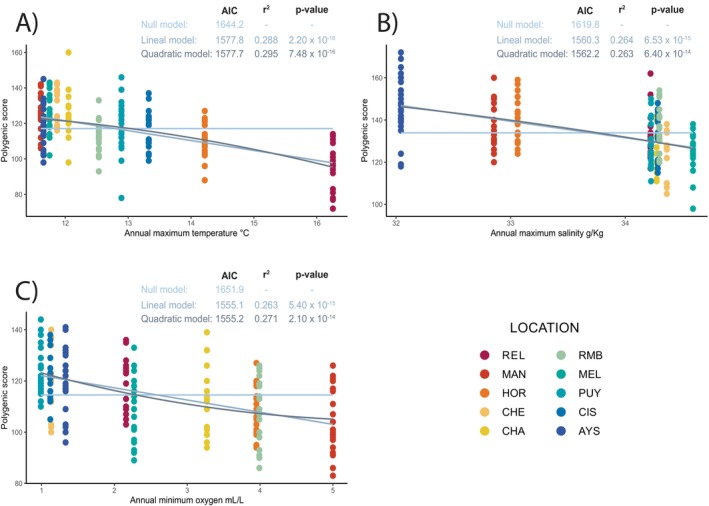
Correlations between additive polygenic scores (APS) based on (A) annual maximum temperature, (B) annual maximum salinity and (C) annual minimum oxygen and 131 putative adaptive loci. Correlation coefficient (*R* ^2^) and *p*‐values and AIC are presented for each variable.

The correlations between additive polygenic scores (APS) and environmental variables were all significant (*p* < 0.001). The *R* ^2^ values ranged from 0.29 for maximum temperature to 0.26 for both maximum salinity and minimum oxygen concentration. APS decreased with increasing temperature, salinity, and oxygen across all environmental variables (Figure 5A,B). The correlation between APS and environmental variables was best represented by linear models, except for maximum temperature, which fitted better with a quadratic model (Figure 5A,B). The correlations calculated for mean and minimum temperature, salinity, and oxygen concentration yielded significant R2 values (Figures S12–S14).

### 3.5 Gene Ontology of Candidate Genes

The BLASTx analysis of the contigs containing the 131 adaptive loci shared by PGD and GEA against bony fish genomes resulted in significant hits (*E*‐value ≤ 1 × 10^−5^) for 790 fish species. The BLASTx similarity results showed that 51 of the 131 contigs containing adaptive loci corresponded to known proteins in the non‐redundant (NR) database (*E*‐value 1e^−5^), of which 36 contigs were functionally annotated. The functional categorization of the annotated sequences involved biological processes, cellular components, and molecular (Figure S15).

A second BLASTx analysis with all 2425 putative loci detected 512 contigs that were functionally annotated. Of these 512 sequences, three were already known to potentially play a role in local adaptation (Table S5). The sequences of the *SNP 6291_214* and *44791_149* belong to the solute carrier (SLC) family. The SLC genes encode transmembrane transporters for inorganic ions, amino acids, neurotransmitters, sugars, purines, and fatty acids, and other solute substrates (Dorwart et al. 2008). The SNP 42_59 is located in the vtgC (13489) gene, which is a member of the vitellogenins (Vtg) family. In egg‐laying vertebrates such as fish, estrogens activate the hepatic synthesis of vitellogenin (Vtg) (Sumpter and Jobling 1995; Brian et al. 2008).

## 4 Discussion

This study provides comprehensive evidence for genomic signatures of local adaptation of *Eleginops maclovinus* to heterogeneous environmental conditions in Northern Patagonia. We conducted a seascape genomics study using 11,961 RAD‐sequencing markers and detected 2164 putative adaptive loci through population genetic differentiation (PGD) and genotype‐environment association (GEA) approaches. Using 131 loci shared by PGD and GEA, we observed that the putative adaptive loci collectively exhibit a highly significant association with various environmental variables, indicating polygenic selection. Additionally, we found contrasting patterns of genetic variation in neutral and adaptive loci that reinforce the possible local adaptation among high levels of gene flow. Subsequently, we detected one gene related to thermal adaptation and two genes that play a role in osmoregulation. In the following, we describe how our study contributes to understanding the role of environmental heterogeneity as a driver of spatial patterns of putative adaptive genetic variation that could influence local adaptation in *E. maclovinus* . Furthermore, we discuss methodological issues about software for detecting putative adaptive loci. Finally, we propose groups based on adaptive divergence for creating management units.

### 4.1 Evidence for Spatially Divergent Selection

Our results indicate spatially divergent selection in *E. maclovinus* , with putative adaptive loci associated with key environmental variables such as temperature, salinity, oxygen, and nutrient concentrations. These associations are supported by prior physiological and ecological studies of the species and provide evidence that environmental heterogeneity in Northern Patagonia may be driving local adaptation.

We detected one gene related to thermal adaptation, consistent with previous findings indicating thermal sensitivity in *E. maclovinus* . Juveniles tolerate temperatures between 4°C and 20°C (Vanella et al. 2012; Oyarzún et al. 2018, 2021) but short‐term exposure to 4°C reduces fitness (Vanella et al. 2012), while long‐term exposure to 15°C–20°C increases metabolism without adverse effects. Temperatures above 25°C negatively impact physiology, growth, and energy substrates balance (Oyarzún et al. 2021). Spawning in spring (Brickle et al. 2005) suggests that elevated temperatures may accelerate maturation, although their effects on early developmental stages remain unclear. Temperature also influences reproductive traits such as egg mortality, embryonic development, and larval metabolism (Pauly and Pullin 1988; Blaxter 1992; Rombough 1997; Van Der Kraak and Pankhurst 1997; Gagliano et al. 2007; Hossain Aunkor et al. 2025). Additionally, temperature may play a role in sexual inversion in protandrous hermaphrodites (Baroiller et al. 1999). While this phenomenon has not been demonstrated in *E. maclovinus* , studies in *Lates calcarifer* show that elevated temperatures increase gonadal aromatase levels, leading to a higher proportion of females (Athauda et al. 2012). These mechanisms, though speculative for *E. maclovinus* , can be taken as a research question for future studies.

Salinity also appears to be a key selective force, as supported by two candidate genes associated with osmoregulation. The *E. maclovinus* juveniles are considered euryhaline, tolerating salinities from 5 to 45 PSU (Vargas‐Chacoff et al. 2014; Vargas‐Chacoff, Moneva, et al. 2015). A reduced trypsin/chymotrypsin (T/C) ratio under low salinity suggests improved food utilization and energy allocation for physiological processes, though osmoregulation in low salinity may reduce growth (Vargas‐Chacoff, Saavedra, et al. 2015). While euryhaline fish have general osmoregulatory capabilities (Laverty and Skadhauge 2012), climate change and anthropogenic pressure may exacerbate salinity stress, posing new challenges for local adaptation (Kültz 2015). In particular, Northern Patagonia is expected to experience reduced precipitation and increased temperature, which may alter salinity regimes (Aguayo et al. 2019), impacting reproduction through DNA damage, sperm motility, egg activation, and DNA recombination (Wai‐sum et al. 2006; Yanagimachi et al. 2017; Hu et al. 2024; Gutierrez et al. 2025).

In addition to temperature and salinity, our results indicate that oxygen availability may be a selective pressure. Oxygen concentration variability can drive anatomical, behavioral, and physiological adaptations for oxygen acquisition and delivery (Nikinmaa and Rees 2005). Fish have evolved mechanisms to balance oxygen transport with the need to avoid toxicity from reactive oxygen species, with hemoglobin diversity reflecting adaptation to low‐oxygen environments (Nikinmaa and Rees 2005; Taylor and McElwain 2010; Dong et al. 2011). *E. maclovinus* hemoglobin (Hb1Em) exhibits higher O_2_ affinity than that of Antarctic notothenioids, suggesting differences in temperature sensitivity (Coppola et al. 2012). Furthermore, the presence of hypoxic zones in the fjords of Northern Patagonia (1.1–2.0 mL/L) highlights the need to investigate the mechanisms underlying *E. maclovinus* adaptation to low‐oxygen conditions, which remains poorly understood.

Finally, we identified putative adaptive loci associated with nutrient concentrations, particularly nitrate, phosphate, and silicate. While these nutrients may not directly exert selection, they likely act as proxies for primary productivity, which can affect food availability and thus exert indirect selective pressures. Patagonian fjords experience seasonal cycles of productivity, with spring–summer blooms of diatoms and pulsed phytoplankton events, and a less productive winter season dominated by smaller phytoplankton (Iriarte et al. 2007; Czypionka et al. 2011; Montero et al. 2011; Paredes and Montecino 2011). Nutrient availability, particularly nitrate and phosphate, limits phytoplankton growth during the productive season (Pizarro et al. 2000; Iriarte et al. 2007). In contrast, increased freshwater discharge in the non‐productive season elevates concentrations of silicic acid and dissolved organic matter, affecting light attenuation (Montero et al. 2017). Previous studies have linked GEA signals in *E. maclovinus* to chlorophyll concentrations as proxies for productivity (Canales‐Aguirre et al. 2022), consistent with similar findings in other marine fishes (Diopere et al. 2017; Cayuela et al. 2020; Maselko et al. 2020; Boulanger et al. 2022).

### 4.2 Additive Polygenic Scores (APS)

For the additive polygenic scores (APS), we observed a strong correlation between APS and maximum temperature and salinity, and minimum oxygen. This suggests local adaptation in *E. maclovinus* populations through polygenic architecture. These findings align with the previously discussed impacts of these environmental variables on fish survival and development (See Section 4.1).

Negative correlations between individual APS and environmental variables suggest directional selection, with different alleles maintained across environmental gradients. The relationship between APS and maximum temperature was slightly better represented by a quadratic model; however, the AIC values for the linear and quadratic models were nearly identical (linear = 1577.8 vs. quadratic = 1577.7), indicating that the data do not strongly discriminate between the two. If the relationship is linear, it would suggest a continuous selective pressure along the temperature gradient. In contrast, a quadratic relationship would point to stabilizing selection at intermediate temperatures. Given the minimal model difference, both interpretations remain plausible and warrant further investigation.

Temperature impacts on additive genetic variation align with adaptive population structure, where the REL population in warmer northern latitudes separates from others. For salinity and oxygen, linear models better explain APS relationships, indicating consistent selection along these gradients. These results are consistent with the observed adaptive population structure, where the CHE and CHA populations differentiate from the rest, with the CHE population exposed to higher oceanic salinities. Lastly, minimum oxygen concentration is critical for reproduction and development, as hypoxia can delay embryonic development and hatching (Richards 2009; Wu 2009). However, the effects of hypoxia on genetic expression and reproductive development in *E. maclovinus* remain unknown.

While the APS approach is valuable for assessing the cumulative effects of adaptive loci in complex environments, it has limitations. Without a reference genome, the possibility of linked adaptive loci cannot be excluded. An annotated *E. maclovinus* genome would enable identification of matches between adaptive loci and genomic regions, clarifying specific genes involved in local adaptation (Manel et al. 2016). Additionally, linking genotypic variation in adaptive loci with individual phenotypic data is essential to establish genotype–phenotype–fitness relationships (Holliday 1969; Blaxter 1992; Brickle et al. 2005; Wu 2009; Lehtonen and Kvarnemo 2015). Our results suggest that maximum temperature, maximum salinity, and minimum oxygen concentration drive spatially variable selection in *E. maclovinus* populations. These factors influence the environmental gradient experienced by eggs and larvae (Holliday 1969; Blaxter 1992; Brickle et al. 2005; Wu 2009; Lehtonen and Kvarnemo 2015), potentially shaping local adaptation. Future research should focus on detecting molecular signatures of selection within a multilocus quantitative genetics framework to better understand the genetic architecture of polygenic adaptive traits.

### 4.3 Finding Functional Genomics of Candidate Genes for Local Adaptation

Using BLASTx and gene ontology, we identified 2425 potentially adaptive loci, with 131 shared by PGD and GEA. Only 512 and 36 loci were annotated, respectively, and just 39.69% of the 131 shared loci matched known sequences, underscoring RAD‐seq limitations without a reference genome (Davey et al. 2013; Andrews et al. 2016; Catchen et al. 2017; McKinney, Larson, et al. 2017). Both datasets revealed loci linked to life‐history traits, such as thermal adaptation and osmoregulation.

We identified five genes linked to life‐history traits: *opa1*, *Stx16*, *FMVIA*, *foxj1b*, and *Lamc3*. The gene *opa1* regulates mitochondrial dynamics and induces apoptosis, which is associated with kidney injury (Song et al. 2017; Yingjie et al. 2019; Herkenne et al. 2020; Wang et al. 2020; Cui et al. 2021; Li et al. 2022). *Stx16* is involved in inducible defense structures in *Daphnia pulex* under predation stress (Spanier et al. 2010). *FMVIA* may play a role in retinal motility in striped bass (Breckler et al. 2000). *foxj1b* is crucial for normal laterality in zebrafish embryos, affecting heart and organ positioning (Tian et al. 2009). Finally, *Lamc3* contributes to parachordal chain development in zebrafish (Eve and Smith 2017).

Expanding our search to adaptive loci, we identified three key genes linked to adaptation: *vtg3* (temperature adaptation) and *slc39a6*, *slc8a2a* (salinity adaptation). *vtg3*, a minor but crucial vitellogenin (Yilmaz et al. 2018, 2019), supports zygote and embryo development and may play a role in thermal adaptation, as seen in *Salmo trutta* (Körner et al. 2008). *slc39a6* maintains zinc homeostasis, vital for cellular growth, and is upregulated in low salinity in *Takifugu rubripes* (Jiang et al. 2020). *slc8a2a*, part of the SLC8 family, regulates Ca^2+^ excretion in seawater fish kidneys and is upregulated during seawater acclimation (Liao et al. 2007; Islam et al. 2011). These genes suggest positive selection for temperature and salinity adaptation in *E. maclovinus* in Northern Patagonia.

We did not identify genes whose functions are supposed to be affected by the concentration of oxygen or nutrients (nitrate, phosphate and silicate). However, many outlier sequences did not have BLASTx results and may represent important proteins that have not been adequately annotated in fish to date.

### 4.4 Comparing Approaches for Detecting Genomic Signal of Selection

The 10 programs used to detect adaptive loci yielded varying results due to differences in algorithms and assumptions. Combining PGD and GEA approaches reduces false positives and enhances detection of selection signals. GEA identified more adaptive loci (2164) than PGD (392), consistent with its greater power to detect divergent selection using multiple environmental variables (de Villemereuil et al. 2014). Within PGD, *fsthet* detected the most loci (313), leveraging empirical *F*_ST_‐heterozygosity distributions without assuming specific evolutionary models (Flanagan and Jones 2017). However, *fsthet* may underperform with low migration rates, which was not an issue here due to high gene flow in *E. maclovinus* (Canales‐Aguirre et al. 2022). *Arlequin* identified 270 loci but has higher error rates in complex scenarios (Narum and Hess 2011). *PCAdapt* was more conservative (15 loci), while *BayeScan* and *OutFLANK* detected none, likely due to admixture and population structure (Luu et al. 2017). Within GEA, *LFMM* detected the most loci (1785), balancing power and error rates, though false positives are possible due to demographic processes (Frichot et al. 2013; de Villemereuil et al. 2014). *MSOD* detected fewer loci (377) but is robust to spatial autocorrelation (Wagner and Dray 2015; Wagner et al. 2017). *RDA* detected the fewest loci (83) due to its multivariate approach but has lower false‐positive rates (Forester et al. 2018; Capblancq et al. 2018; Capblancq and Forester 2021). *BayeScEnv* and *Samβada* detected no loci, with limited comparative studies available. Considering that *BayeScEnv* produces fewer candidate loci than *BayeScan* (de Villemereuil and Gaggiotti 2015), it is to be expected that if *BayeScan* did not detect any loci, *BayeScEnv* would not either. Combining PGD and GEA approaches is crucial to minimize errors and improve detection of adaptive loci in complex scenarios.

### 4.5 Implications for Management and Conservation

The 11,961 filtered loci genotyped for 202 individuals (the largest population genomic data set reported so far for *Eleginops maclovinus* ) offers high resolution for creating management policies and decisions with respect to patterns of adaptive genetic variation. Although neutral loci did not reveal a clear genetic structure among locations, analysis of 131 shared adaptive loci indicated genetic differentiation separating the populations into four groups: REL, CHE, CHA, and a fourth group comprising the remaining seven sampling locations. Note that all neutral pairwise *F*_ST_ values were close to zero, similar to that shown by Canales‐Aguirre et al. (2022) and also from fish and other marine species (Nayfa and Zenger 2016; Asaduzzaman, Wahab, et al. 2019; Asaduzzaman, Igarashi, et al. 2019; O'Leary et al. 2021). The interpretation of extremely small *F*_ST_ is not straightforward (Waples and Gaggiotti 2006; Conover et al. 2006; Waples et al. 2008), meaning it is unclear what the implications of the patterns of weak neutral differentiation may be for demographic connectivity of subpopulations. Nevertheless, the signals of putatively adaptive genetic differentiation between the 10 locations were significant. Our study demonstrates how the environment shapes the distribution of adaptive genetic variation across space for *E. maclovinus* , showing that REL, CHE, and CHA retain adaptive characteristics different from the rest. This can have important implications for prioritizing sites for protection, where the level of adaptive genetic variation could be an indicator of the evolutionary resilience of populations (Bonin et al. 2007; Sgrò et al. 2011; Crozier and Siegel 2025; Ahi et al. 2025). Moreover, understanding the spatial distribution of putatively adaptive genetic variation can inform the selection of specific sites for protection within marine reserves to maintain the adaptive potential and evolutionary resilience of wild populations faced with environmental change (von der Heyden 2017; Logez et al. 2025). The adaptive divergence reported here appears to be underpinned by a variation in seawater temperature and oxygen concentrations in Northern Patagonia (Iriarte et al. 2014; Torres et al. 2014; Pérez‐Santos et al. 2014; Silva and Vargas 2014) and should affect traits relevant for the survival, growth, and reproduction of *E. maclovinus* . The complex patterns of population structure and putatively adaptive diversity among *E. maclovinus* locations that we showed here are not recognized in current *E. maclovinus* management units. To date, there is no management plan for *E. maclovinus* in Chile, only regulations for the type of fishing gear used. Future research could use neutral and adaptive datasets to determine management units using adaptive (e.g., population adaptive index, adaptive score) and neutral (e.g., expected heterozygosity, local differentiation) metrics as suggested by Xuereb et al. (2021). In the Chilean Northern Patagonia, temperature and heat content are predicted to increase, and freshwater river inputs are predicted to decrease due to climate change (Aguayo et al. 2019). As such, the spatial patterns of genetic variability observed in this study can inform conservation planning decisions by identifying for protection populations containing higher levels of segregating polymorphisms associated with environmental conditions (e.g., temperature and salinity) that are prone to change in the future.

## Author Contributions

**C. Eliza Claure:** data curation (equal), formal analysis (equal), visualization (equal), writing – original draft (equal), writing – review and editing (equal). **Garrett J. McKinney:** investigation (equal), methodology (equal), software (equal), writing – review and editing (equal). **José Dellis Rocha:** data curation (equal), methodology (equal), software (equal), writing – review and editing (equal). **José M. Yáñez:** investigation (equal), supervision (equal), writing – original draft (equal), writing – review and editing (equal). **Iván Pérez‐Santos:** data curation (equal), investigation (equal), software (equal), visualization (equal), writing – original draft (equal). **Cristian B. Canales‐Aguirre:** conceptualization (lead), data curation (equal), funding acquisition (lead), investigation (equal), project administration (lead), resources (equal), supervision (lead), writing – original draft (equal), writing – review and editing (equal).

## Conflicts of Interest

The authors declare no conflicts of interest.

## Supporting information


Data S1

## Data Availability

The data used for this study are available at https://github.com/Canales‐AguirreCB/Claure_AdaptiveGenetics_MS.

## References

[ece371524-bib-0001] Aguayo, R. , J. León‐Muñoz , J. Vargas‐Baecheler , et al. 2019. “The Glass Half‐Empty: Climate Change Drives Lower Freshwater Input in the Coastal System of the Chilean Northern Patagonia.” Climatic Change 155: 417–435.

[ece371524-bib-0003] Ahi, E. P. , A. S. Lindeza , A. Miettinen , and C. R. Primmer . 2025. “Transcriptional Responses to Changing Environments: Insights From Salmonids.” Reviews in Fish Biology and Fisheries 35.

[ece371524-bib-0005] Andrews, K. R. , J. M. Good , M. R. Miller , G. Luikart , and P. A. Hohenlohe . 2016. “Harnessing the Power of RADseq for Ecological and Evolutionary Genomics.” Nature Reviews Genetics 17: 81–92. 10.1038/nrg.2015.28 PMC4823021 26729255

[ece371524-bib-0006] Andrews, K. R. , T. Seaborn , J. P. Egan , et al. 2023. “Whole Genome Resequencing Identifies Local Adaptation Associated With Environmental Variation for Redband Trout.” Molecular Ecology 32: 800–818. 36478624 10.1111/mec.16810 PMC9905331

[ece371524-bib-0007] Aracena, C. , C. B. Lange , J. L. Iriarte , L. Rebolledo , and S. Pantoja . 2011. “Latitudinal Patterns of Export Production Recorded in Surface Sediments of the Chilean Patagonian Fjords (41–55 S) as a Response to Water Column Productivity.” Continental Shelf Research 31: 340–355.

[ece371524-bib-0008] Araneda, C. , M. A. Larraín , B. Hecht , and S. Narum . 2016. “Adaptive Genetic Variation Distinguishes Chilean Blue Mussels (*Mitilus chilensis*) From Different Marine Environments.” Ecology and Evolution 6: 3632–3644. 27195104 10.1002/ece3.2110 PMC4851556

[ece371524-bib-0009] Asaduzzaman, M. , Y. Igarashi , M. A. Wahab , et al. 2019. “Population Genomics of an Anadromous Hilsa Shad *Tenualosa ilisha* Species Across Its Diverse Migratory Habitats: Discrimination by Fine‐Scale Local Adaptation.” Genes 11, no. 1: 46. 31905942 10.3390/genes11010046 PMC7017241

[ece371524-bib-0010] Asaduzzaman, M. , M. A. Wahab , M. J. Rahman , et al. 2019. “Fine‐Scale Population Structure and Ecotypes of Anadromous Hilsa Shad (*Tenualosa ilisha*) Across Complex Aquatic Ecosystems Revealed by NextRAD Genotyping.” Scientific Reports 9: 16050. 31690767 10.1038/s41598-019-52465-2 PMC6831668

[ece371524-bib-0011] Assis, J. , L. Tyberghein , and S. Bosch . 2018. “Bio‐ORACLE v2. 0: Extending Marine Data Layers for Bioclimatic Modelling.” Global Ecology and Biogeography 27: 277–284.

[ece371524-bib-0012] Athauda, S. , T. Anderson , and R. de Nys . 2012. “Effect of Rearing Water Temperature on Protandrous Sex Inversion in Cultured Asian Seabass (*Lates calcarifer*).” General and Comparative Endocrinology 175: 416–423. 22155035 10.1016/j.ygcen.2011.11.040

[ece371524-bib-0013] Babin, C. , P.‐A. Gagnaire , S. A. Pavey , and L. Bernatchez . 2017. “RAD‐Seq Reveals Patterns of Additive Polygenic Variation Caused by Spatially‐Varying Selection in the American Eel (*Anguilla rostrata*).” Genome Biology and Evolution 9: 2974–2986. 29136139 10.1093/gbe/evx226 PMC5714190

[ece371524-bib-0015] Baird, N. A. , P. D. Etter , T. S. Atwood , et al. 2008. “Rapid SNP Discovery and Genetic Mapping Using Sequenced RAD Markers.” PLoS One 3: e3376. 18852878 10.1371/journal.pone.0003376 PMC2557064

[ece371524-bib-0016] Baroiller, J.‐F. , Y. Guiguen , and A. Fostier . 1999. “Endocrine and Environmental Aspects of Sex Differentiation in Fish.” Cellular and Molecular Life Sciences 55: 910–931.

[ece371524-bib-0018] Beaumont, M. A. 2005. “Adaptation and Speciation: What Can Fst Tell Us?” Trends in Ecology & Evolution 20: 435–440. 16701414 10.1016/j.tree.2005.05.017

[ece371524-bib-0020] Bekkevold, D. , J. Höjesjö , E. E. Nielsen , et al. 2020. “Northern European *Salmo trutta* (L.) Populations Are Genetically Divergent Across Geographical Regions and Environmental Gradients.” Evolutionary Applications 13: 400–416. 31993085 10.1111/eva.12877 PMC6976966

[ece371524-bib-0023] Bernatchez, L. 2016. “On the Maintenance of Genetic Variation and Adaptation to Environmental Change: Considerations From Population Genomics in Fishes.” Journal of Fish Biology 89: 2519–2556. 27687146 10.1111/jfb.13145

[ece371524-bib-0024] Blaxter, J. H. S. 1992. “The Effect of Temperature on Larval Fishes.” Netherlands Journal of Zoology 42: 336–357.

[ece371524-bib-0025] Bonin, A. , F. Nicole , F. Pompanon , C. Miaud , and P. Taberlet . 2007. “Population Adaptive Index: A New Method to Help Measure Intraspecific Genetic Diversity and Prioritize Populations for Conservation.” Conservation Biology 21: 697–708. 17531048 10.1111/j.1523-1739.2007.00685.x

[ece371524-bib-0026] Boulanger, E. , L. Benestan , P.‐E. Guerin , A. Dalongeville , D. Mouillot , and S. Manel . 2022. “Climate Differently Influences the Genomic Patterns of Two Sympatric Marine Fish Species.” Journal of Animal Ecology 91: 1180–1195. 34716929 10.1111/1365-2656.13623

[ece371524-bib-0027] Breckler, J. , K. Au , J. Cheng , T. Hasson , and B. Burnside . 2000. “Novel Myosin VI Isoform Is Abundantly Expressed in Retina.” Experimental Eye Research 70: 121–134. 10644428 10.1006/exer.1999.0758

[ece371524-bib-0028] Brian, J. V. , C. A. Harris , T. J. Runnalls , et al. 2008. “Evidence of Temperature‐Dependent Effects on the Estrogenic Response of Fish: Implications With Regard to Climate Change.” Science of the Total Environment 397: 72–81. 18423818 10.1016/j.scitotenv.2008.02.036

[ece371524-bib-0029] Brickle, P. , V. Laptikhovsky , and A. Arkhipkin . 2005. “Reproductive Strategy of a Primitive Temperate Notothenioid *Eleginops maclovinus* .” Journal of Fish Biology 66: 1044–1059.

[ece371524-bib-0030] Canales‐Aguirre, C. B. , S. Ferrada‐Fuentes , R. Galleguillos , and C. E. Hernández . 2016. “Genetic Structure in a Small Pelagic Fish Coincides With a Marine Protected Area: Seascape Genetics in Patagonian Fjords.” PLoS One 11: e0160670. 27505009 10.1371/journal.pone.0160670 PMC4978504

[ece371524-bib-0031] Canales‐Aguirre, C. B. , S. Ferrada‐Fuentes , R. Galleguillos , F. X. Oyarzun , C. C. Buratti , and C. E. Hernández . 2018. “High Genetic Diversity and Low‐Population Differentiation in the Patagonian Sprat (*Sprattus fuegensis*) Based on Mitochondrial DNA.” Mitochondrial DNA Part A, DNA Mapping, Sequencing, and Analysis 29: 1148–1155. 29334843 10.1080/24701394.2018.1424841

[ece371524-bib-0032] Canales‐Aguirre, C. B. , W. A. Larson , G. J. McKinney , et al. 2022. “Neutral and Adaptive Loci Reveal Fine‐Scale Population Structure in *Eleginops maclovinus* From North Patagonia.” Ecology and Evolution 12: e9343. 36225825 10.1002/ece3.9343 PMC9530513

[ece371524-bib-0033] Capblancq, T. , and B. R. Forester . 2021. “Redundancy Analysis: A Swiss Army Knife for Landscape Genomics.” Methods in Ecology and Evolution 12: 2298–2309.

[ece371524-bib-0034] Capblancq, T. , K. Luu , M. G. B. Blum , and E. Bazin . 2018. “Evaluation of Redundancy Analysis to Identify Signatures of Local Adaptation.” Molecular Ecology Resources 18: 1223–1233. 29802785 10.1111/1755-0998.12906

[ece371524-bib-0035] Catchen, J. M. , P. A. Hohenlohe , L. Bernatchez , W. C. Funk , K. R. Andrews , and F. W. Allendorf . 2017. “Unbroken: RADseq Remains a Powerful Tool for Understanding the Genetics of Adaptation in Natural Populations.” Molecular Ecology Resources 17: 362–365. 28319339 10.1111/1755-0998.12669

[ece371524-bib-0037] Cayuela, H. , Q. Rougemont , M. Laporte , et al. 2020. “Shared Ancestral Polymorphisms and Chromosomal Rearrangements as Potential Drivers of Local Adaptation in a Marine Fish.” Molecular Ecology 29: 2379–2398. 32497342 10.1111/mec.15499

[ece371524-bib-0038] Ceballos, S. G. , E. P. Lessa , R. Licandeo , and D. A. Fernández . 2016. “Genetic Relationships Between Atlantic and Pacific Populations of the Notothenioid Fish *Eleginops maclovinus*: The Footprints of Quaternary Glaciations in Patagonia.” Heredity 116: 372–377. 26696136 10.1038/hdy.2015.106 PMC4806693

[ece371524-bib-0039] Ceballos, S. G. , E. P. Lessa , M. F. Victorio , and D. A. Fernández . 2012. “Phylogeography of the Sub‐Antarctic Notothenioid Fish *Eleginops maclovinus*: Evidence of Population Expansion.” Marine Biology 159: 499–505.

[ece371524-bib-0040] Chen, L. , Y. Lu , W. Li , et al. 2019. “The Genomic Basis for Colonizing the Freezing Southern Ocean Revealed by Antarctic Toothfish and Patagonian Robalo Genomes.” GigaScience 8: giz016. 30715292 10.1093/gigascience/giz016 PMC6457430

[ece371524-bib-0041] Chen, Z. , C.‐H. C. Cheng , J. Zhang , et al. 2008. “Transcriptomic and Genomic Evolution Under Constant Cold in Antarctic Notothenioid Fish.” Proceedings of the National Academy of Sciences of the United States of America 105: 12944–12949. 18753634 10.1073/pnas.0802432105 PMC2529033

[ece371524-bib-0043] Conesa, A. , and S. Götz . 2008. “Blast2GO: A Comprehensive Suite for Functional Analysis in Plant Genomics.” International Journal of Plant Genomics 2008: 619832. 18483572 10.1155/2008/619832 PMC2375974

[ece371524-bib-0044] Conover, D. O. , L. M. Clarke , S. B. Munch , and G. N. Wagner . 2006. “Spatial and Temporal Scales of Adaptive Divergence in Marine Fishes and the Implications for Conservation.” Journal of Fish Biology 69: 21–47.

[ece371524-bib-0045] Coppola, D. , S. Abbruzzetti , F. Nicoletti , et al. 2012. “ATP Regulation of the Ligand‐Binding Properties in Temperate and Cold‐Adapted Haemoglobins. X‐Ray Structure and Ligand‐Binding Kinetics in the Sub‐Antarctic Fish *Eleginops maclovinus* .” Molecular BioSystems 8: 3295–3304. 23086282 10.1039/c2mb25210d

[ece371524-bib-0047] Crozier, L. G. , and J. E. Siegel . 2025. “From Threats to Solutions: A Literature Review of Climate Adaptation in Anadromous Salmon and Trout.” Ecosphere 16: e70054.

[ece371524-bib-0048] Cui, Y. , K. Yin , Y. Zheng , et al. 2021. “Mixed Plasticizers Aggravated Apoptosis by NOD2‐RIP2‐NF‐κB Pathway in Grass Carp Hepatocytes.” Journal of Hazardous Materials 402: 123527. 32712359 10.1016/j.jhazmat.2020.123527

[ece371524-bib-0049] Cuvier, G. , and A. Valenciennes . 1830. Histoire naturelle des poissons. F.‐G. Levrault.

[ece371524-bib-0050] Czypionka, T. , C. A. Vargas , N. Silva , G. Daneri , H. E. González , and J. L. Iriarte . 2011. “Importance of Mixotrophic Nanoplankton in Aysén Fjord (Southern Chile) During Austral Winter.” Continental Shelf Research 31: 216–224.

[ece371524-bib-0053] Davey, J. W. , T. Cezard , P. Fuentes‐Utrilla , C. Eland , K. Gharbi , and M. L. Blaxter . 2013. “Special Features of RAD Sequencing Data: Implications for Genotyping.” Molecular Ecology 22: 3151–3164. 23110438 10.1111/mec.12084 PMC3712469

[ece371524-bib-0054] Dávila, P. M. , D. Figueroa , and E. Müller . 2002. “Freshwater Input Into the Coastal Ocean and Its Relation With the Salinity Distribution off Austral Chile (35–55° S).” Continental Shelf Research 22: 521–534.

[ece371524-bib-0055] de Villemereuil, P. , É. Frichot , É. Bazin , O. François , and O. E. Gaggiotti . 2014. “Genome Scan Methods Against More Complex Models: When and How Much Should We Trust Them?” Molecular Ecology 23: 2006–2019. 24611968 10.1111/mec.12705

[ece371524-bib-0056] de Villemereuil, P. , and O. E. Gaggiotti . 2015. “A New F_ST_‐Based Method to Uncover Local Adaptation Using Environmental Variables.” Methods in Ecology and Evolution 6: 1248–1258.

[ece371524-bib-0057] Delgado, M. L. , K. Górski , E. Habit , and D. E. Ruzzante . 2019. “The Effects of Diadromy and Its Loss on Genomic Divergence: The Case of Amphidromous *Galaxias maculatus* Populations.” Molecular Ecology 28: 5217–5231. 31652382 10.1111/mec.15290

[ece371524-bib-0061] Diopere, E. , S. G. Vandamme , P. I. Hablützel , et al. 2017. “Seascape Genetics of a Flatfish Reveals Local Selection Under High Levels of Gene Flow.” ICES Journal of Marine Science 75: 675–689.

[ece371524-bib-0062] Dong, X. Y. , J. G. Qin , and X. M. Zhang . 2011. “Fish Adaptation to Oxygen Variations in Aquaculture From Hypoxia to Hyperoxia.” African Journal of Fisheries and Aquaculture 2: 23.

[ece371524-bib-0063] Dorwart, M. R. , N. Shcheynikov , D. Yang , and S. Muallem . 2008. “The Solute Carrier 26 Family of Proteins in Epithelial Ion Transport.” Physiology 23: 104–114. 18400693 10.1152/physiol.00037.2007

[ece371524-bib-0064] Dowling, D. K. , and L. W. Simmons . 2009. “Reactive Oxygen Species as Universal Constraints in Life‐History Evolution.” Proceedings of the Royal Society B: Biological Sciences 276: 1737–1745. 10.1098/rspb.2008.1791 PMC2674489 19324792

[ece371524-bib-0067] Duruz, S. , N. Sevane , O. Selmoni , et al. 2019. “Rapid Identification and Interpretation of Gene‐Environment Associations Using the New R.SamBada Landscape Genomics Pipeline.” Molecular Ecology Resources 19: 1355–1365. 31136078 10.1111/1755-0998.13044 PMC6790591

[ece371524-bib-0068] Eastman, J. T. 1993. Antarctic Fish Biology: Evolution in a Unique Environment. Academic Press.

[ece371524-bib-0069] Evanno, G. , S. Regnaut , and J. Goudet . 2005. “Detecting the Number of Clusters of Individuals Using the Software STRUCTURE: A Simulation Study.” Molecular Ecology 14: 2611–2620. 15969739 10.1111/j.1365-294X.2005.02553.x

[ece371524-bib-0070] Eve, A. M. J. , and J. C. Smith . 2017. “Knockdown of Laminin Gamma‐3 (Lamc3) Impairs Motoneuron Guidance in the Zebrafish Embryo.” Wellcome Open Research 2: 111. 29417095 10.12688/wellcomeopenres.12394.1 PMC5785718

[ece371524-bib-0071] Excoffier, L. , and H. E. L. Lischer . 2010. “Arlequin Suite ver 3.5: A New Series of Programs to Perform Population Genetics Analyses Under Linux and Windows.” Molecular Ecology Resources 10: 564–567. 21565059 10.1111/j.1755-0998.2010.02847.x

[ece371524-bib-0074] Flanagan, S. P. , and A. G. Jones . 2017. “Constraints on the FST‐Heterozygosity Outlier Approach.” Journal of Heredity 108: 561–573. 28486592 10.1093/jhered/esx048

[ece371524-bib-0076] Foll, M. , and O. Gaggiotti . 2008. “A Genome‐Scan Method to Identify Selected Loci Appropriate for Both Dominant and Codominant Markers: A Bayesian Perspective.” Genetics 180: 977–993. 18780740 10.1534/genetics.108.092221 PMC2567396

[ece371524-bib-0077] Forester, B. R. , J. R. Lasky , H. H. Wagner , and D. L. Urban . 2018. “Comparing Methods for Detecting Multilocus Adaptation With Multivariate Genotype‐Environment Associations.” Molecular Ecology 27: 2215–2233. 29633402 10.1111/mec.14584

[ece371524-bib-0079] Francis, R. M. 2017. “Pophelper: An R Package and Web App to Analyse and Visualize Population Structure.” Molecular Ecology Resources 17: 27–32. 26850166 10.1111/1755-0998.12509

[ece371524-bib-0080] François, O. , H. Martins , K. Caye , and S. D. Schoville . 2016. “Controlling False Discoveries in Genome Scans for Selection.” Molecular Ecology 25: 454–469. 26671840 10.1111/mec.13513

[ece371524-bib-0083] Frichot, E. , S. D. Schoville , G. Bouchard , and O. François . 2013. “Testing for Associations Between Loci and Environmental Gradients Using Latent Factor Mixed Models.” Molecular Biology and Evolution 30: 1687–1699. 23543094 10.1093/molbev/mst063 PMC3684853

[ece371524-bib-0084] Fuentes‐Pardo, A. P. , R. Stanley , C. Bourne , et al. 2024. “Adaptation to Seasonal Reproduction and Environment‐Associated Factors Drive Temporal and Spatial Differentiation in Northwest Atlantic Herring Despite Gene Flow.” Evolutionary Applications 17: e13675. 38495946 10.1111/eva.13675 PMC10940790

[ece371524-bib-0085] Gagliano, M. , M. I. McCormick , and M. G. Meekan . 2007. “Temperature‐Induced Shifts in Selective Pressure at a Critical Developmental Transition.” Oecologia 152: 219–225. 17242907 10.1007/s00442-006-0647-1

[ece371524-bib-0086] Gagnaire, P.‐A. , T. Broquet , D. Aurelle , et al. 2015. “Using Neutral, Selected, and Hitchhiker Loci to Assess Connectivity of Marine Populations in the Genomic Era.” Evolutionary Applications 8: 769–786. 26366195 10.1111/eva.12288 PMC4561567

[ece371524-bib-0087] Gagnaire, P.‐A. , and O. E. Gaggiotti . 2016. “Detecting Polygenic Selection in Marine Populations by Combining Population Genomics and Quantitative Genetics Approaches.” Current Zoology 62: 603–616. 29491948 10.1093/cz/zow088 PMC5804256

[ece371524-bib-0090] González, H. E. , L. Castro , G. Daneri , et al. 2011. “Seasonal Plankton Variability in Chilean Patagonia Fjords: Carbon Flow Through the Pelagic Food Web of Aysen Fjord and Plankton Dynamics in the Moraleda Channel Basin.” Continental Shelf Research 31: 225–243.

[ece371524-bib-0091] Gosztonyi, A. E. , and G. Ae . 1974. “Edad y crecimiento del “Róbalo” *Eleginops maclovinus* (Osteichthyes, Nototheniidae) en aguas de la ría Deseado y sus adyacencias.”

[ece371524-bib-0092] Goudet, J. 2005. “Hierfstat, a Package for r to Compute and Test Hierarchical F‐Statistics.” Molecular Ecology Notes 5: 184–186.

[ece371524-bib-0094] Gutierrez, M. F. , V. S. Andrade , A. Ale , et al. 2025. “Responses of Freshwater Organisms to Multiple Stressors in a Climate Change Scenario: A Review on Small‐Scale Experiments.” Environmental Science and Pollution Research 32: 4431–4444. 39903437 10.1007/s11356-025-36034-x

[ece371524-bib-0096] Hair, J. F. , R. E. Anderson , R. L. Tatham , and W. C. Black . 1995. Multivariate Data Analysis With Readings. Prentice Hall.

[ece371524-bib-0098] Herkenne, S. , O. Ek , M. Zamberlan , et al. 2020. “Developmental and Tumor Angiogenesis Requires the Mitochondria‐Shaping Protein Opa1.” Cell Metabolism 31: 987–1003.e8. 32315597 10.1016/j.cmet.2020.04.007

[ece371524-bib-0099] Hoban, S. , M. Bruford , J. D'Urban Jackson , et al. 2020. “Genetic Diversity Targets and Indicators in the CBD Post‐2020 Global Biodiversity Framework Must Be Improved.” Biological Conservation 248: 108654.

[ece371524-bib-0100] Hoban, S. , J. L. Kelley , K. E. Lotterhos , et al. 2016. “Finding the Genomic Basis of Local Adaptation: Pitfalls, Practical Solutions, and Future Directions.” American Naturalist 188: 379–397. 10.1086/688018 PMC5457800 27622873

[ece371524-bib-0102] Holliday, F. 1969. “4 the Effects of Salinity on the Eggs and Larvae of Teleosts.” Fish Physiology 1: 293–311.

[ece371524-bib-0103] Hossain Aunkor, M. T. , M. M. Hasan Khan , M. A. Kabir , M. T. Raihan , and M. F. Miah . 2025. “The Heat Shock Matters With Specific Attention to the Expression of Hsp60 Under Thermal Stress in Fish: A Critical Review.” Ecological Genetics and Genomics 35: 100337.

[ece371524-bib-0105] Hu, J. , J. Yang , and H. Liao . 2024. “Progress on Stress Resistance Breeding in Fish.” Reproduction and Breeding 4: 267–278.

[ece371524-bib-0106] Iriarte, J. L. , H. E. González , K. K. Liu , C. Rivas , and C. Valenzuela . 2007. “Spatial and Temporal Variability of Chlorophyll and Primary Productivity in Surface Waters of Southern Chile (41.5–43 S).” Estuarine, Coastal and Shelf Science 74: 471–480.

[ece371524-bib-0107] Iriarte, J. L. , H. E. González , and L. Nahuelhual . 2010. “Patagonian Fjord Ecosystems in Southern Chile as a Highly Vulnerable Region: Problems and Needs.” Ambio 39: 463–466. 21090000 10.1007/s13280-010-0049-9 PMC3357667

[ece371524-bib-0108] Iriarte, J. L. , S. Pantoja , and G. Daneri . 2014. “Oceanographic Processes in Chilean Fjords of Patagonia: From Small to Large‐Scale Studies.” Progress in Oceanography 129: 1–7.

[ece371524-bib-0109] Islam, Z. , A. Kato , M. F. Romero , and S. Hirose . 2011. “Identification and Apical Membrane Localization of an Electrogenic Na^+^/Ca^2+^ Exchanger NCX2a Likely to Be Involved in Renal Ca^2+^ Excretion by Seawater Fish.” American Journal of Physiology. Regulatory, Integrative and Comparative Physiology 301: R1427–R1439. 21880864 10.1152/ajpregu.00165.2011 PMC3213929

[ece371524-bib-0110] Jacob, B. G. , F. J. Tapia , G. Daneri , et al. 2014. “Springtime Size‐Fractionated Primary Production Across Hydrographic and PAR‐Light Gradients in Chilean Patagonia (41–50° S).” Progress in Oceanography 129: 75–84.

[ece371524-bib-0114] Jiang, J.‐L. , J. Xu , L. Ye , et al. 2020. “Identification of Differentially Expressed Genes in Gills of Tiger Puffer (*Takifugu rubripes*) in Response to Low‐Salinity Stress.” Comparative Biochemistry and Physiology Part B, Biochemistry & Molecular Biology 243‐244: 110437. 10.1016/j.cbpb.2020.110437 32247057

[ece371524-bib-0115] Jombart, T. 2008. “adegenet: A R Package for the Multivariate Analysis of Genetic Markers.” Bioinformatics 24: 1403–1405. 18397895 10.1093/bioinformatics/btn129

[ece371524-bib-0116] Jombart, T. , and I. Ahmed . 2011. “adegenet 1.3‐1: New Tools for the Analysis of Genome‐Wide SNP Data.” Bioinformatics 27: 3070–3071. 21926124 10.1093/bioinformatics/btr521 PMC3198581

[ece371524-bib-0117] Jombart, T. , S. Devillard , and F. Balloux . 2010. “Discriminant Analysis of Principal Components: A New Method for the Analysis of Genetically Structured Populations.” BMC Genetics 11: 94. 20950446 10.1186/1471-2156-11-94 PMC2973851

[ece371524-bib-0119] Khrustaleva, A. M. 2024. “SNP Polymorphisms Are Associated With Environmental Factors in Sockeye Salmon Populations Across the Northwest Pacific: Insights From Redundancy Analysis.” Genes 15: 1485. 39596685 10.3390/genes15111485 PMC11593481

[ece371524-bib-0120] Kock, K.‐H. 1992. Antarctic Fish and Fisheries. Cambridge University Press.

[ece371524-bib-0121] Körner, O. , S. Kohno , R. Schönenberger , et al. 2008. “Water Temperature and Concomitant Waterborne Ethinylestradiol Exposure Affects the Vitellogenin Expression in Juvenile Brown Trout (*Salmo trutta*).” Aquatic Toxicology 90: 188–196. 18947890 10.1016/j.aquatox.2008.08.012

[ece371524-bib-0122] Kültz, D. 2015. “Physiological Mechanisms Used by Fish to Cope With Salinity Stress.” Journal of Experimental Biology 218: 1907–1914. 26085667 10.1242/jeb.118695

[ece371524-bib-0123] Laikre, L. , S. Hoban , M. W. Bruford , et al. 2020. “Post‐2020 Goals Overlook Genetic Diversity.” Science (New York, N.Y.) 367: 1083–1085. 10.1126/science.abb2748 32139534

[ece371524-bib-0124] Lattuca, M. E. , C. C. Boy , F. A. Vanella , M. E. Barrantes , and D. A. Fernández . 2018. “Thermal Responses of Three Native Fishes From Estuarine Areas of the Beagle Channel, and Their Implications for Climate Change.” Hydrobiologia 808: 235–249.

[ece371524-bib-0125] Laverty, G. , and E. Skadhauge . 2012. “Adaptation of Teleosts to Very High Salinity.” Comparative Biochemistry and Physiology. Part A, Molecular & Integrative Physiology 163: 1–6. 10.1016/j.cbpa.2012.05.203 22640831

[ece371524-bib-0127] Lehtonen, T. K. , and C. Kvarnemo . 2015. “Density Effects on Fish Egg Survival and Infections Depend on Salinity.” Marine Ecology Progress Series 540: 183–191.

[ece371524-bib-0128] Lex, A. , N. Gehlenborg , H. Strobelt , R. Vuillemot , and H. Pfister . 2014. “UpSet: Visualization of Intersecting Sets.” IEEE Transactions on Visualization and Computer Graphics 20: 1983–1992. 26356912 10.1109/TVCG.2014.2346248 PMC4720993

[ece371524-bib-0129] Li, J. , W. Zhang , P. Zhou , X. Tong , D. Guo , and H. Lin . 2022. “Selenium Deficiency Induced Apoptosis via Mitochondrial Pathway Caused by Oxidative Stress in Porcine Gastric Tissues.” Research in Veterinary Science 144: 142–148. 34809980 10.1016/j.rvsc.2021.10.017

[ece371524-bib-0130] Li, Y.‐L. , D.‐X. Xue , B.‐D. Zhang , and J.‐X. Liu . 2019. “Population Genomic Signatures of Genetic Structure and Environmental Selection in the Catadromous Roughskin Sculpin *Trachidermus fasciatus* .” Genome Biology and Evolution 11: 1751–1764. 31173074 10.1093/gbe/evz118 PMC6601870

[ece371524-bib-0131] Liao, B.‐K. , A.‐N. Deng , S.‐C. Chen , M.‐Y. Chou , and P.‐P. Hwang . 2007. “Expression and Water Calcium Dependence of Calcium Transporter Isoforms in Zebrafish Gill Mitochondrion‐Rich Cells.” BMC Genomics 8: 354. 17915033 10.1186/1471-2164-8-354 PMC2140269

[ece371524-bib-0132] Linford, P. , I. Pérez‐Santos , P. Montero , et al. 2024. “Oceanographic Processes Driving Low‐Oxygen Conditions Inside Patagonian Fjords.” Biogeosciences 21: 1433–1459.

[ece371524-bib-0133] Linford, P. , I. Pérez‐Santos , P. Montero , et al. 2023a. “Oceanographic Processes Favoring Deoxygenation Inside Patagonian Fjords.”

[ece371524-bib-0134] Linford, P. , I. Pérez‐Santos , I. Montes , et al. 2023b. “Recent Deoxygenation of Patagonian Fjord Subsurface Waters Connected to the Peru–Chile Undercurrent and Equatorial Subsurface Water Variability.” Global Biogeochemical Cycles 37: e2022GB007688.

[ece371524-bib-0135] Lischer, H. E. L. , and L. Excoffier . 2012. “PGDSpider: An Automated Data Conversion Tool for Connecting Population Genetics and Genomics Programs.” Bioinformatics 28: 298–299. 22110245 10.1093/bioinformatics/btr642

[ece371524-bib-0136] Logez, M. , L. Bouraï , N. Hette‐Tronquart , and C. Argillier . 2025. “Ecological Vulnerability of Aquatic Ecosystems – A Review.” Environmental Management 75: 192–204. 39510995 10.1007/s00267-024-02076-z

[ece371524-bib-0137] Luu, K. , E. Bazin , and M. G. B. Blum . 2017. “Pcadapt: An R Package to Perform Genome Scans for Selection Based on Principal Component Analysis.” Molecular Ecology Resources 17: 67–77. 27601374 10.1111/1755-0998.12592

[ece371524-bib-0138] Manel, S. , C. Perrier , M. Pratlong , et al. 2016. “Genomic Resources and Their Influence on the Detection of the Signal of Positive Selection in Genome Scans.” Molecular Ecology 25: 170–184. 26562485 10.1111/mec.13468

[ece371524-bib-0139] Marquet, P. A. , A. H. Buschmann , D. Corcoran , et al. 2023. “Global Change and Acceleration of Anthropic Pressures on Patagonian Ecosystems.” In Integrated Science, 33–65. Springer International Publishing.

[ece371524-bib-0140] Maselko, J. , K. R. Andrews , and P. A. Hohenlohe . 2020. “Long‐Lived Marine Species May Be Resilient to Environmental Variability Through a Temporal Portfolio Effect.” Ecology and Evolution 10: 6435–6448. 32724524 10.1002/ece3.6378 PMC7381576

[ece371524-bib-0141] McKinney, G. , M. V. McPhee , C. Pascal , J. E. Seeb , and L. W. Seeb . 2020. “Network Analysis of Linkage Disequilibrium Reveals Genome Architecture in Chum Salmon.” G3: Genes, Genomes, Genetics 10: 1553–1561. 32165371 10.1534/g3.119.400972 PMC7202013

[ece371524-bib-0142] McKinney, G. J. , W. A. Larson , L. W. Seeb , and J. E. Seeb . 2017. “RADseq Provides Unprecedented Insights Into Molecular Ecology and Evolutionary Genetics: Comment on Breaking RAD by Lowry Et al. (2016).” Molecular Ecology Resources 17: 356–361. 28028934 10.1111/1755-0998.12649

[ece371524-bib-0143] McKinney, G. J. , R. K. Waples , L. W. Seeb , and J. E. Seeb . 2017. “Paralogs Are Revealed by Proportion of Heterozygotes and Deviations in Read Ratios in Genotyping‐By‐Sequencing Data From Natural Populations.” Molecular Ecology 17: 656–669. 10.1111/1755-0998.12613 27762098

[ece371524-bib-0144] Miller, M. R. , J. P. Dunham , A. Amores , and W. A. Cresko . 2007. “Rapid and Cost‐Effective Polymorphism Identification and Genotyping Using Restriction Site Associated DNA (RAD) Markers.” Genome Research 17: 240–248. 17189378 10.1101/gr.5681207 PMC1781356

[ece371524-bib-0145] Montero, P. , G. Daneri , H. E. González , et al. 2011. “Seasonal Variability of Primary Production in a Fjord Ecosystem of the Chilean Patagonia: Implications for the Transfer of Carbon Within Pelagic Food Webs.” Continental Shelf Research 31: 202–215.

[ece371524-bib-0146] Montero, P. , G. Daneri , F. Tapia , J. L. Iriarte , and D. Crawford . 2017. “Diatom Blooms and Primary Production in a Channel Ecosystem of Central Patagonia.” Latin American Journal of Aquatic Research 45: 999–1016.

[ece371524-bib-0147] Narum, S. R. , and J. E. Hess . 2011. “Comparison of F(ST) Outlier Tests for SNP Loci Under Selection.” Molecular Ecology Resources 11, no. Suppl 1: 184–194. 21429174 10.1111/j.1755-0998.2011.02987.x

[ece371524-bib-0148] Nayfa, M. G. , and K. R. Zenger . 2016. “Unravelling the Effects of Gene Flow and Selection in Highly Connected Populations of the Silver‐Lip Pearl Oyster (*Pinctada maxima*).” Marine Genomics 28: 99–106. 26934995 10.1016/j.margen.2016.02.005

[ece371524-bib-0149] Nikinmaa, M. , and B. B. Rees . 2005. “Oxygen‐Dependent Gene Expression in Fishes.” American Journal of Physiology. Regulatory, Integrative and Comparative Physiology 288: R1079–R1090. 15821280 10.1152/ajpregu.00626.2004

[ece371524-bib-0150] North, A. W. 2001. “Early Life History Strategies of Notothenioids at South Georgia.” Journal of Fish Biology 58: 496–505.

[ece371524-bib-0151] Oksanen, J. , F. Guillaume , and M. Blanchet . 2016. “vegan: Community Ecology Package.”

[ece371524-bib-0153] O'Leary, S. J. , C. M. Hollenbeck , R. R. Vega , and D. S. Portnoy . 2021. “Disentangling Complex Genomic Signals to Understand Population Structure of an Exploited, Estuarine‐Dependent Flatfish.” Ecology and Evolution 11: 13415–13429. 34646479 10.1002/ece3.8064 PMC8495835

[ece371524-bib-0154] Oyarzún, R. , J. L. P. Muñoz , J. P. Pontigo , F. J. Morera , and L. Vargas‐Chacoff . 2018. “Effects of Acclimation to High Environmental Temperatures on Intermediary Metabolism and Osmoregulation in the Sub‐Antarctic Notothenioid *Eleginops maclovinus* .” Marine Biology 165: 1–15.

[ece371524-bib-0155] Oyarzún, R. , J. J. Rojas , J. P. Pontigo , et al. 2021. “Long‐Term Effects of Temperatures on the Physiological Response of Juveniles of the Eurythermal Sub‐Antarctic Notothenioid *Eleginops maclovinus* .” Aquaculture 530: 1–11.

[ece371524-bib-0156] Palumbi, S. R. 2003. “Population Genetics, Demographic Connectivity, and the Design of Marine Reserves.” Ecological Applications 13: 146–158.

[ece371524-bib-0158] Pantoja, S. , J. Iriarte , and G. Daneri . 2011. “Oceanography of the Chilean Patagonia.” Continental Shelf Research 31: 149–153.

[ece371524-bib-0159] Paredes, A. , and V. Montecino . 2011. “Size Diversity as an Expression of Phytoplankton Community Structure and the Identification of Its Patterns on the Scale of Fjords and Channels.” Continental Shelf Research 31: 272–281.

[ece371524-bib-0160] Pascual, M. , B. Rives , C. Schunter , and E. Macpherson . 2017. “Impact of Life History Traits on Gene Flow: A Multispecies Systematic Review Across Oceanographic Barriers in the Mediterranean Sea.” PLoS One 12: e0176419. 28489878 10.1371/journal.pone.0176419 PMC5425013

[ece371524-bib-0161] Pauls, S. U. , C. Nowak , M. Bálint , and M. Pfenninger . 2013. “The Impact of Global Climate Change on Genetic Diversity Within Populations and Species.” Molecular Ecology 22: 925–946. 23279006 10.1111/mec.12152

[ece371524-bib-0162] Pauly, D. , and R. S. V. Pullin . 1988. “Hatching Time in Spherical, Pelagic, Marine Fish Eggs in Response to Temperature and Egg Size.” Environmental Biology of Fishes 22: 261–271.

[ece371524-bib-0163] Pembleton, L. W. , N. O. I. Cogan , and J. W. Forster . 2013. “StAMPP: An R Package for Calculation of Genetic Differentiation and Structure of Mixed‐Ploidy Level Populations.” Molecular Ecology Resources 13: 946–952. 23738873 10.1111/1755-0998.12129

[ece371524-bib-0164] Pequeño, G. H. 1989. “The Geographical Distribution and Taxonomic Arrangement of South American Notothenidae Fishes (Osteichthyes, Notothenidae).” Boletín de la Sociedad de Biología de Concepción, Chile 60: 183–200.

[ece371524-bib-0165] Pérez‐Santos, I. , J. Garcés‐Vargas , W. Schneider , L. Ross , S. Parra , and A. Valle‐Levinson . 2014. “Double‐Diffusive Layering and Mixing in Patagonian Fjords.” Progress in Oceanography 129: 35–49.

[ece371524-bib-0166] Pizarro, G. , J. L. Iriarte , V. Montecino , J. L. Blanco , and L. Guzmán . 2000. “Distribución de la biomasa fitoplanctónica y productividad primaria máxima de fiordos y canales australes (47–50 S) en octubre 1996.” Ciencia y Tecnología del Mar.

[ece371524-bib-0168] Pritchard, J. K. , M. Stephens , and P. Donnelly . 2000. “Inference of Population Structure Using Multilocus Genotype Data.” Genetics 155: 945–959. 10835412 10.1093/genetics/155.2.945 PMC1461096

[ece371524-bib-0169] Privé, F. , K. Luu , B. J. Vilhjálmsson , and M. G. B. Blum . 2020. “Performing Highly Efficient Genome Scans for Local Adaptation With R Package pcadapt Version 4.” Molecular Biology and Evolution 37: 2153–2154. 32343802 10.1093/molbev/msaa053

[ece371524-bib-0170] Rabassa, J. , A. Coronato , and O. Martínez . 2011. “Late Cenozoic Glaciations in Patagonia and Tierra del Fuego: An Updated Review.” Biological Journal of the Linnean Society. Linnean Society of London 103: 316–335.

[ece371524-bib-0171] Rellstab, C. , F. Gugerli , A. J. Eckert , A. M. Hancock , and R. Holderegger . 2015. “A Practical Guide to Environmental Association Analysis in Landscape Genomics.” Molecular Ecology 24: 4348–4370. 26184487 10.1111/mec.13322

[ece371524-bib-0172] Richards, J. G. 2009. “Chapter 10 Metabolic and Molecular Responses of Fish to Hypoxia.” In Fish Physiology, edited by J. G. Richards , A. P. Farrell , and C. J. Brauner , 443–485. Academic Press.

[ece371524-bib-0173] Rodrigo, C. 2008. “Submarine Topography in the Chilean North Patagonian Channels. Progress in the Oceanographic Knowledge of Chilean Inner Waters, From Puerto Montt to Cape Horn.” Comité Oceanográfico Nacional‐Pontificia Universidad Católica de Valparaíso, Valparaíso, 19–23.

[ece371524-bib-0174] Rombough, P. J. 1997. “The Effects of Temperature on Embryonic and Larval Development.” In Global Warming: Implications for Freshwater and Marine Fish, 177–224. Cambridge University Press.

[ece371524-bib-0177] Schlitzer, R. 2002. “Interactive Analysis and Visualization of Geoscience Data With Ocean Data View.” Computers & Geosciences 28: 1211–1218.

[ece371524-bib-0178] Schneider, W. , D. Donoso , J. Garcés‐Vargas , and R. Escribano . 2017. “Water‐Column Cooling and Sea Surface Salinity Increase in the Upwelling Region Off Central‐South Chile Driven by a Poleward Displacement of the South Pacific High.” Progress in Oceanography 151: 38–48.

[ece371524-bib-0179] Schneider, W. , I. Pérez‐Santos , L. Ross , L. Bravo , R. Seguel , and F. Hernández . 2014. “On the Hydrography of Puyuhuapi Channel, Chilean Patagonia.” Progress in Oceanography 129: 8–18.

[ece371524-bib-0181] Segovia, N. I. , C. A. González‐Wevar , and P. A. Haye . 2020. “Signatures of Local Adaptation in the Spatial Genetic Structure of the Ascidian *Pyura chilensis* Along the Southeast Pacific Coast.” Scientific Reports 10: 14098. 32839518 10.1038/s41598-020-70798-1 PMC7445245

[ece371524-bib-0182] Sgrò, C. M. , A. J. Lowe , and A. A. Hoffmann . 2011. “Building Evolutionary Resilience for Conserving Biodiversity Under Climate Change.” Evolutionary Applications 4: 326–337. 25567976 10.1111/j.1752-4571.2010.00157.x PMC3352557

[ece371524-bib-0184] Silva, C. , I. Andrade , E. Yáñez , et al. 2016. “Predicting Habitat Suitability and Geographic Distribution of Anchovy (*Engraulis ringens*) due to Climate Change in the Coastal Areas Off Chile.” Progress in Oceanography 146: 159–174.

[ece371524-bib-0185] Silva, N. , and C. A. Vargas . 2014. “Hypoxia in Chilean Patagonian Fjords.” Progress in Oceanography 129: 62–74.

[ece371524-bib-0187] Song, X.‐B. , G. Liu , F. Liu , et al. 2017. “Autophagy Blockade and Lysosomal Membrane Permeabilization Contribute to Lead‐Induced Nephrotoxicity in Primary Rat Proximal Tubular Cells.” Cell Death & Disease 8: e2863. 28594408 10.1038/cddis.2017.262 PMC5520918

[ece371524-bib-0189] Spanier, K. I. , F. Leese , C. Mayer , et al. 2010. “Predator‐Induced Defences in *Daphnia pulex*: Selection and Evaluation of Internal Reference Genes for Gene Expression Studies With Real‐Time PCR.” BMC Molecular Biology 11: 50. 20587017 10.1186/1471-2199-11-50 PMC3148505

[ece371524-bib-0190] Stucki, S. , P. Orozco‐terWengel , B. R. Forester , et al. 2017. “High Performance Computation of Landscape Genomic Models Including Local Indicators of Spatial Association.” Molecular Ecology Resources 17: 1072–1089. 27801969 10.1111/1755-0998.12629

[ece371524-bib-0191] Sumpter, J. P. , and S. Jobling . 1995. “Vitellogenesis as a Biomarker for Estrogenic Contamination of the Aquatic Environment.” Environmental Health Perspectives 103, no. Suppl 7: 173–178. 10.1289/ehp.95103s7173 PMC1518861 8593867

[ece371524-bib-0192] Taylor, C. T. , and J. C. McElwain . 2010. “Ancient Atmospheres and the Evolution of Oxygen Sensing via the Hypoxia‐Inducible Factor in Metazoans.” Physiology 25: 272–279. 20940432 10.1152/physiol.00029.2010

[ece371524-bib-0194] Tian, T. , L. Zhao , M. Zhang , X. Zhao , and A. Meng . 2009. “Both foxj1a and foxj1b Are Implicated in Left‐Right Asymmetric Development in Zebrafish Embryos.” Biochemical and Biophysical Research Communications 380: 537–542. 19284996 10.1016/j.bbrc.2009.01.111

[ece371524-bib-0195] Torres, R. , N. Silva , B. Reid , and M. Frangopulos . 2014. “Silicic Acid Enrichment of Subantarctic Surface Water From Continental Inputs Along the Patagonian Archipelago Interior Sea (41–56 S).” Progress in Oceanography 129: 50–61.

[ece371524-bib-0197] Tyberghein, L. , H. Verbruggen , K. Pauly , C. Troupin , F. Mineur , and O. De Clerck . 2012. “Bio‐ORACLE: A Global Environmental Dataset for Marine Species Distribution Modelling.” Global Ecology and Biogeography 21: 272–281.

[ece371524-bib-0198] Van Der Kraak, G. , and N. W. Pankhurst . 1997. “Temperature Effects on the Reproductive Performance of Fish.” In Global Warming: Implications for Freshwater and Marine Fish, 159–176. Cambridge University Press.

[ece371524-bib-0199] Vanella, F. A. , C. C. Boy , and D. A. Fernández . 2012. “Temperature Effects on Growing, Feeding, and Swimming Energetics in the Patagonian Blennie *Eleginops maclovinus* (Pisces: Perciformes).” Polar Biology 35: 1861–1868.

[ece371524-bib-0200] Vargas‐Chacoff, L. , F. Moneva , R. Oyarzún , et al. 2014. “Environmental Salinity‐Modified Osmoregulatory Response in the Sub‐Antarctic Notothenioid Fish *Eleginops maclovinus* .” Polar Biology 37: 1235–1245.

[ece371524-bib-0201] Vargas‐Chacoff, L. , F. Moneva , R. Oyarzún , et al. 2015. “Metabolic Responses to Salinity Changes in the Subantarctic Notothenioid Teleost *Eleginops maclovinus* .” Polar Biology 39: 1297–1308.

[ece371524-bib-0202] Vargas‐Chacoff, L. , E. Saavedra , R. Oyarzún , et al. 2015. “Effects on the Metabolism, Growth, Digestive Capacity and Osmoregulation of Juvenile of Sub‐Antarctic Notothenioid Fish *Eleginops maclovinus* Acclimated at Different Salinities.” Fish Physiology and Biochemistry 41: 1369–1381. 26148800 10.1007/s10695-015-0092-3

[ece371524-bib-0203] Venables, W. N. , and B. D. Ripley . 2013. Modern Applied Statistics With S‐PLUS. Springer Science & Business Media.

[ece371524-bib-0204] von der Heyden, S. 2017. “Making Evolutionary History Count: Biodiversity Planning for Coral Reef Fishes and the Conservation of Evolutionary Processes.” Coral Reefs 36: 183–194.

[ece371524-bib-0205] Wagner, H. H. , M. Chávez‐Pesqueira , and B. R. Forester . 2017. “Spatial Detection of Outlier Loci With Moran Eigenvector Maps.” Molecular Ecology Resources 17: 1122–1135. 28067020 10.1111/1755-0998.12653

[ece371524-bib-0206] Wagner, H. H. , and S. Dray . 2015. “Generating Spatially Constrained Null Models for Irregularly Spaced Data Using Moran Spectral Randomization Methods.” Methods in Ecology and Evolution 6: 1169–1178.

[ece371524-bib-0207] Wai‐sum, O. , H. Chen , and P. H. Chow . 2006. “Male Genital Tract Antioxidant Enzymes—Their Ability to Preserve Sperm DNA Integrity.” Molecular and Cellular Endocrinology 250: 80–83. 16442705 10.1016/j.mce.2005.12.029

[ece371524-bib-0209] Wang, Z. , J. Wu , Z. Hu , et al. 2020. “Dexmedetomidine Alleviates Lipopolysaccharide‐Induced Acute Kidney Injury by Inhibiting p75NTR‐Mediated Oxidative Stress and Apoptosis.” Oxidative Medicine and Cellular Longevity 2020: 5454210. 33194004 10.1155/2020/5454210 PMC7648709

[ece371524-bib-0210] Waples, R. S. , and O. Gaggiotti . 2006. “What Is a Population? An Empirical Evaluation of Some Genetic Methods for Identifying the Number of Gene Pools and Their Degree of Connectivity.” Molecular Ecology 15: 1419–1439. 16629801 10.1111/j.1365-294X.2006.02890.x

[ece371524-bib-0211] Waples, R. S. , A. E. Punt , and J. M. Cope . 2008. “Integrating Genetic Data Into Management of Marine Resources: How Can We Do It Better?” Fish and Fisheries 9: 423–449.

[ece371524-bib-0214] Weir, B. S. , and C. C. Cockerham . 1984. “Estimating F‐Statistics for the Analysis of Population Structure.” Evolution; International Journal of Organic Evolution 38: 1358–1370. 28563791 10.1111/j.1558-5646.1984.tb05657.x

[ece371524-bib-0215] Whitlock, M. C. , and K. E. Lotterhos . 2015. “Reliable Detection of Loci Responsible for Local Adaptation: Inference of a Null Model Through Trimming the Distribution of F(ST).” American Naturalist 186, no. Suppl 1: S24–S36. 10.1086/682949 26656214

[ece371524-bib-0216] Wigginton, J. E. , D. J. Cutler , and G. R. Abecasis . 2005. “A Note on Exact Tests of Hardy–Weinberg Equilibrium.” American Journal of Human Genetics 76: 887–893. 15789306 10.1086/429864 PMC1199378

[ece371524-bib-0218] Wu, R. S. S. 2009. “Chapter 3 Effects of Hypoxia on Fish Reproduction and Development.” In Fish Physiology, edited by J. G. Richards , A. P. Farrell , and C. J. Brauner , 79–141. Academic Press.

[ece371524-bib-0219] Xu, Q. , C.‐H. C. Cheng , P. Hu , et al. 2008. “Adaptive Evolution of Hepcidin Genes in Antarctic Notothenioid Fishes.” Molecular Biology and Evolution 25: 1099–1112. 18310660 10.1093/molbev/msn056

[ece371524-bib-0220] Xuereb, A. , C. C. D'Aloia , M. Andrello , L. Bernatchez , and M.‐J. Fortin . 2021. “Incorporating Putatively Neutral and Adaptive Genomic Data Into Marine Conservation Planning.” Conservation Biology: The Journal of the Society for Conservation Biology 35: 909–920. 32785955 10.1111/cobi.13609

[ece371524-bib-0221] Yanagimachi, R. , T. Harumi , H. Matsubara , et al. 2017. “Chemical and Physical Guidance of Fish Spermatozoa Into the Egg Through the Micropyle.” Biology of Reproduction 96, no. 4: 780–799. 28371886 10.1093/biolre/iox015 PMC6355103

[ece371524-bib-0222] Yilmaz, O. , A. Patinote , T. Nguyen , and J. Bobe . 2018. “Multiple Vitellogenins in Zebrafish (*Danio rerio*): Quantitative Inventory of Genes, Transcripts and Proteins, and Relation to Egg Quality.” Fish Physiology and Biochemistry 44: 1509–1525. 29882000 10.1007/s10695-018-0524-y

[ece371524-bib-0223] Yilmaz, O. , A. Patinote , T. Nguyen , E. Com , C. Pineau , and J. Bobe . 2019. “Genome Editing Reveals Reproductive and Developmental Dependencies on Specific Types of Vitellogenin in Zebrafish (*Danio rerio*).” Molecular Reproduction and Development 86: 1168–1188. 31380595 10.1002/mrd.23231

[ece371524-bib-0224] Yingjie, K. , Y. Haihong , C. Lingwei , et al. 2019. “Apoptosis Repressor With Caspase Recruitment Domain Deficiency Accelerates Ischemia/Reperfusion (I/R)‐induced Acute Kidney Injury by Suppressing Inflammation and Apoptosis: The Role of AKT/mTOR Signaling.” Biomedicine & Pharmacotherapy = Biomedecine & Pharmacotherapie 112: 108681. 30970510 10.1016/j.biopha.2019.108681

[ece371524-bib-0226] Zuur, A. F. , E. N. Ieno , and C. S. Elphick . 2010. “A Protocol for Data Exploration to Avoid Common Statistical Problems.” Methods in Ecology and Evolution/British Ecological Society 1: 3–14.

