# Supplementary material for: Genomic Signals of Local Adaptation Associated With Environmental Variables in Eleginops maclovinus From Northern Chilean Patagonia
Source: Ecol Evol. 2025 Jun 24;15(6):e71524. doi: 10.1002/ece3.71524 (PMC12186005; doi:10.1002/ece3.71524)
Supplement: Supplementary file 1 — Data S1 [file ECE3-15-e71524-s001.docx]

**Genomic signals of local adaptation associated with environmental variables in *Eleginops maclovinus* from Northern Chilean** **Patagonia**

C. Eliza Claure ^1,2,3,4^, Garrett J. McKinney ^5^, J. Dellis Rocha ^6^, José M. Yáñez^7^, Iván Pérez-Santos ^1,8,9^, Cristian B. Canales-Aguirre ^1,2^

^1^ Centro i~mar, Universidad de Los Lagos, Camino a Chinquihue 6 km, Puerto Montt, Chile

^2^ Núcleo Milenio INVASAL, Concepción, Chile

^3^ Programa de Magister en Ciencias, mención Producción, Manejo y Conservación de Recursos Naturales, Universidad de Los Lagos

^4^ Programa de Doctorado en Ciencias, mención Conservación y Manejo de Recursos Naturales, Universidad de Los Lagos

^5^ Washington Department of Fish and Wildlife, Seattle, Washington, USA

^6^ Escuela de Obstetricia, Facultad de Ciencias para el Cuidado de la Salud, Universidad San Sebastián Sede de La Patagonia, Puerto Montt, Región de Los Lagos

^7^ Facultad de Ciencias Veterinarias y Pecuarias, Universidad de Chile, Av Santa Rosa 11735, La Pintana, Santiago 8820808, Chile

^8^ Center for Oceanographic Research COPAS Sur-Austral and COPAS COASTAL, Universidad de Concepción, Chile

^9^ Centro de Investigaciones en Ecosistemas de la Patagonia (CIEP), Coyhaique, Chile

*Corresponding author:

Cristian B. Canales-Aguirre

Centro i~mar, Universidad de Los Lagos

Camino a Chinquihue 6 km, Puerto Montt, Chile

[cristian.canales@ulagos.cl](mailto:criastian.canales@ulagos.cl)

**Supplementary data**

**Supplementary Figures**


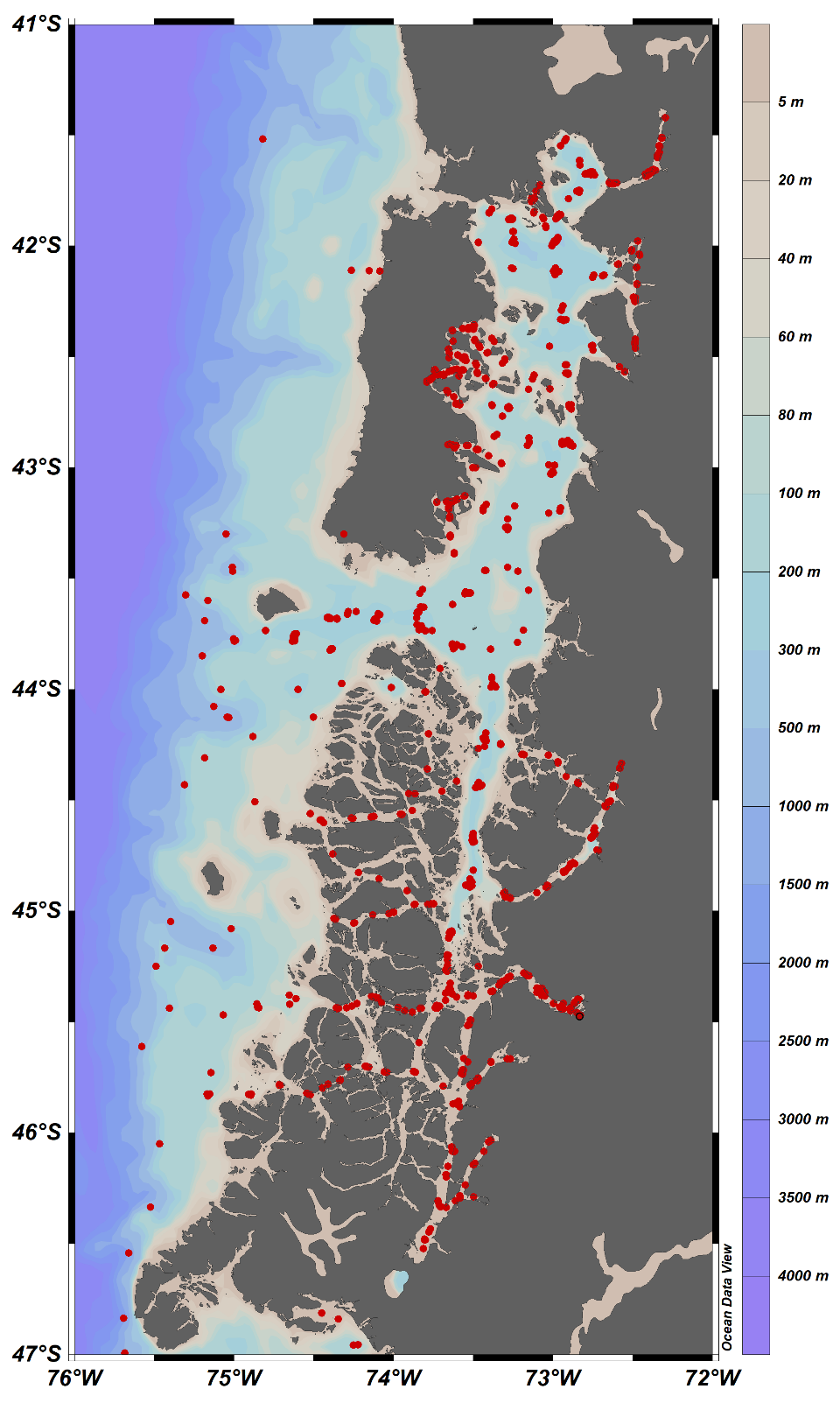


**Figure S1** Distribution of oceanographic station from CIMAR campaigns. Map showing the distribution of stations sampled during oceanographic campaigns CIMAR 1 to CIMAR 24 carried out in Northern Patagonia fjords and channels from.

**
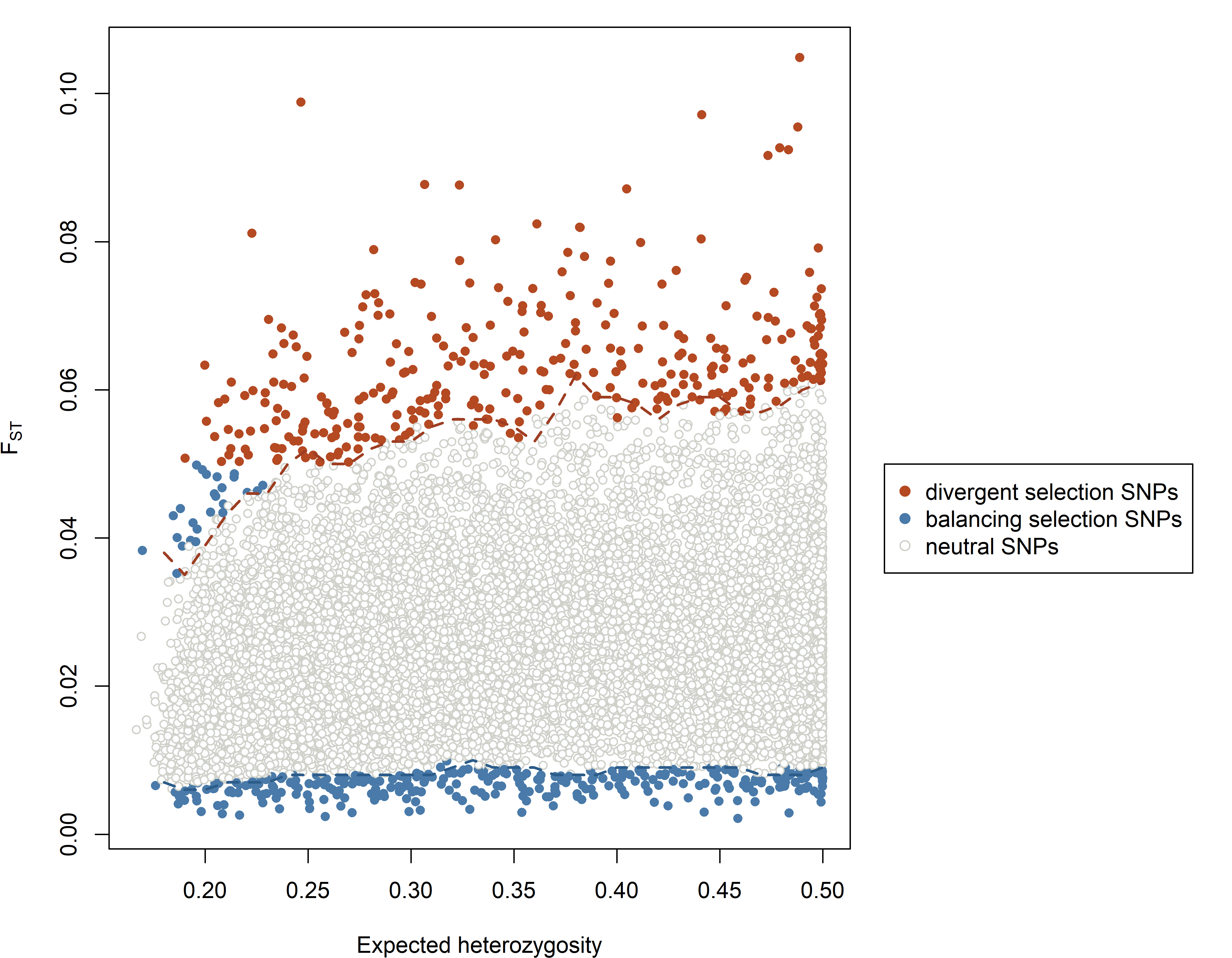
**

**Figure S2** Graph of the distribution of expected heterozygosity values in relation to F_ST_ values. Orange, blue and gray points represent putatively under divergent selection, under balancing selection, and neutral loci respectively using a threshold of 0.05. The lines show 95% smoothed quantiles calculated by *fsthet*.

**
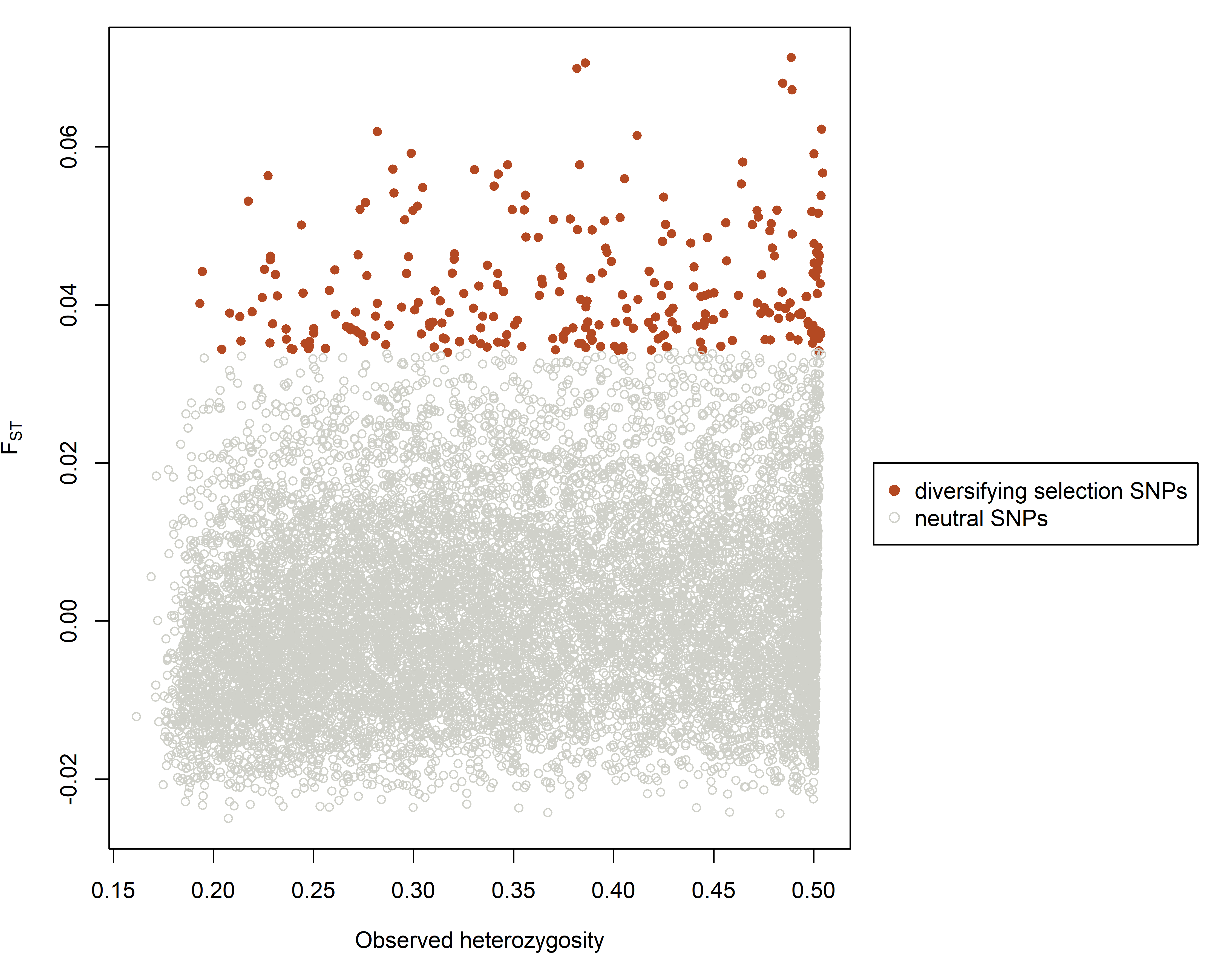
**

**Figure S3** Outlier loci under the hierarchical structure. Model using *Arlequin 3.5.2.2.* are above the 95% quantile: F_ST_ and observed heterozygosity, putatively neutral loci are plotted as gray circles and putative loci under divergent selection are represented as orange circles. The putative loci under balancing selection were not identified by this software.

**
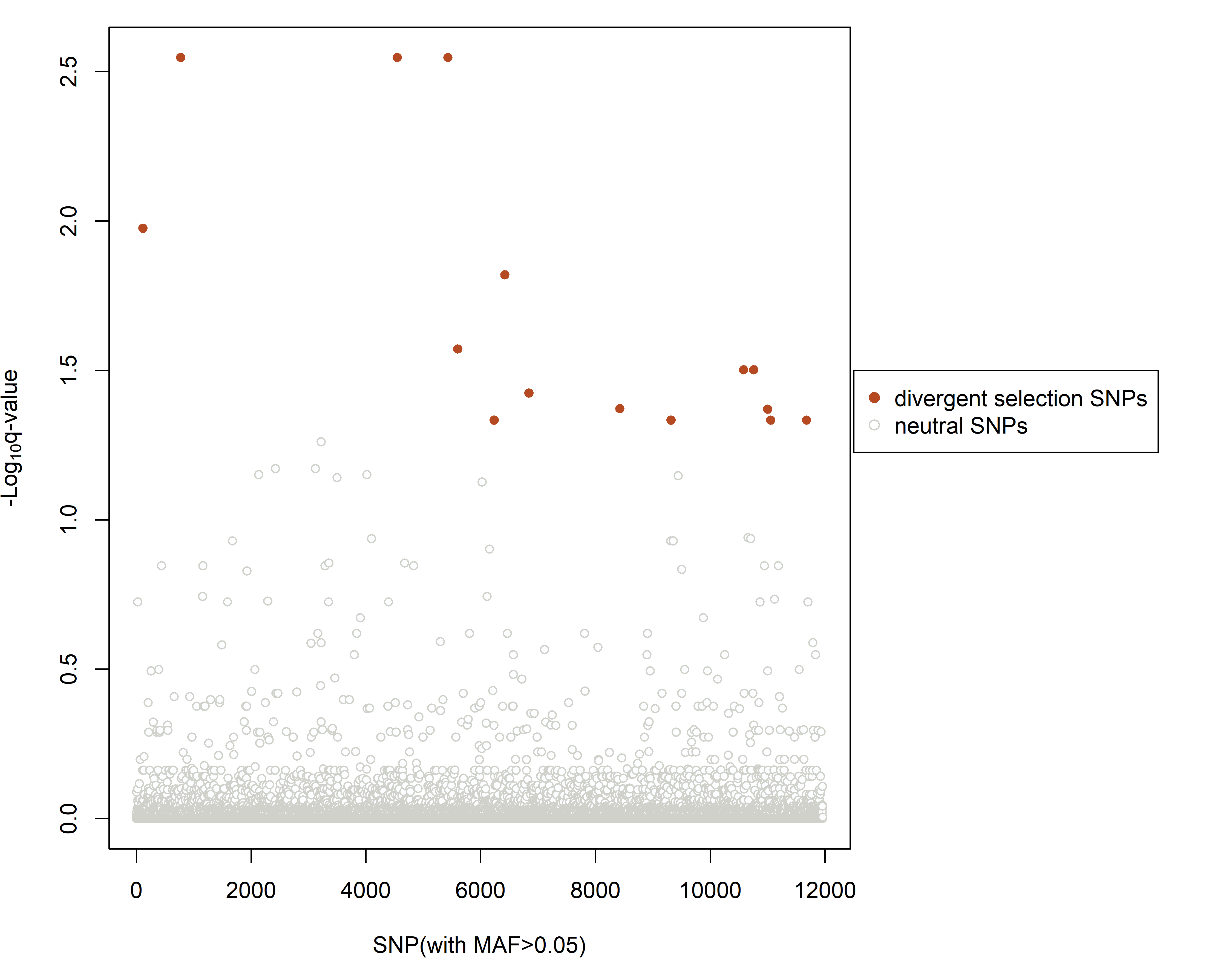
** **Figure S4** Manhattan plot showing the distribution of putative adaptative loci. Those loci obtained from *PCAdapt* where Y-axis represents p-values and putative adaptative loci above α = 0.05 are represented as orange circles.

**
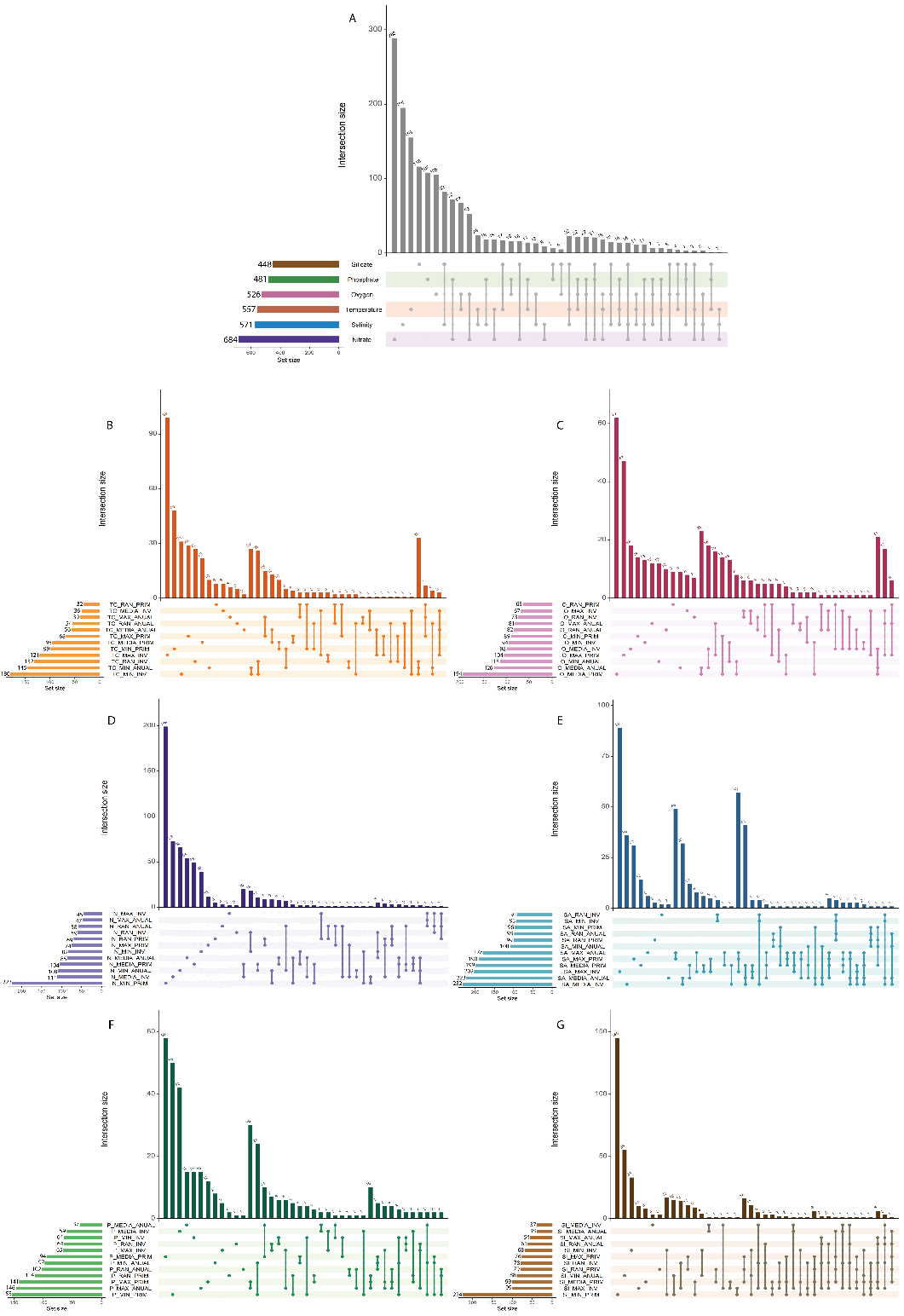
**

**Figure S5** UpSet diagrams for all intersections of adaptative loci detected by *LFMM*. A) Summary of all putative adaptative loci. Disaggregated of results by B) Conservative temperature (ºC), C) Oxygen concentration (mL/L), D) Nitrate concentration (µM), E) Absolute salinity (g/Kg), F) Phosphate concentration (µM) and G) Silicate concentration (µM). The set size (horizontal bars) indicates the total number of loci correlated with that environmental variable. The intersection size (vertical bars) indicates the number of loci per set intersection, the dots represent the set of unique loci correlated only with that environmental variable and no other one, while the dots connected by line represent loci shared by two, three, or more environmental variable.


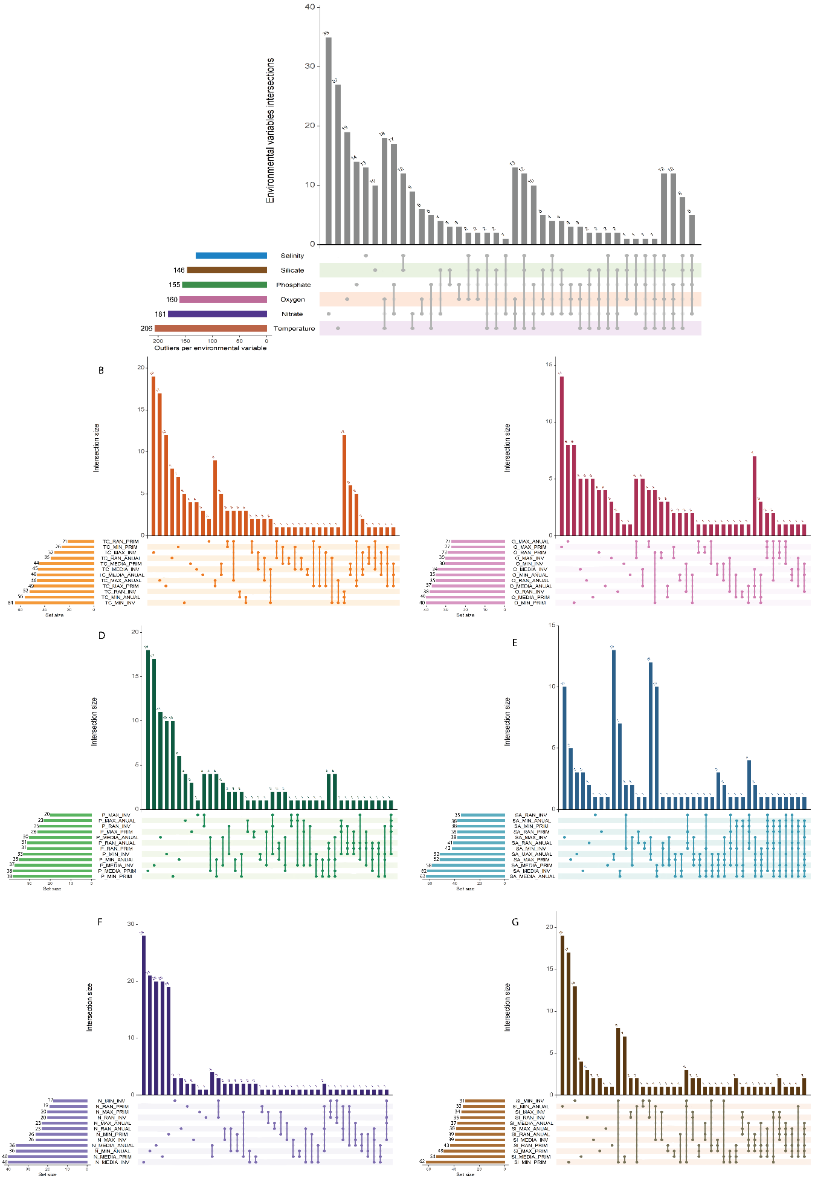


**Figure S6** UpSet diagrams for all intersections of adaptative loci detected by *MSOD.*

A) Summary of all putative adaptative loci. Disaggregated of results by B) Conservative temperature (ºC) C) Oxygen concentration (mL/L), D) Nitrate concentration (µM), E) Absolute salinity (g/Kg), F) Phosphate concentration (µM) and G) Silicate (µM) concentration. The set size (horizontal bars) indicates the total number of loci correlated with that environmental variable. The intersection size (vertical bars) indicates the number of loci per set intersection, the dots represent the set of unique loci correlated only with that environmental variable and no other one, while the dots connected by line represent loci shared by two, three, or more environmental variable.

**
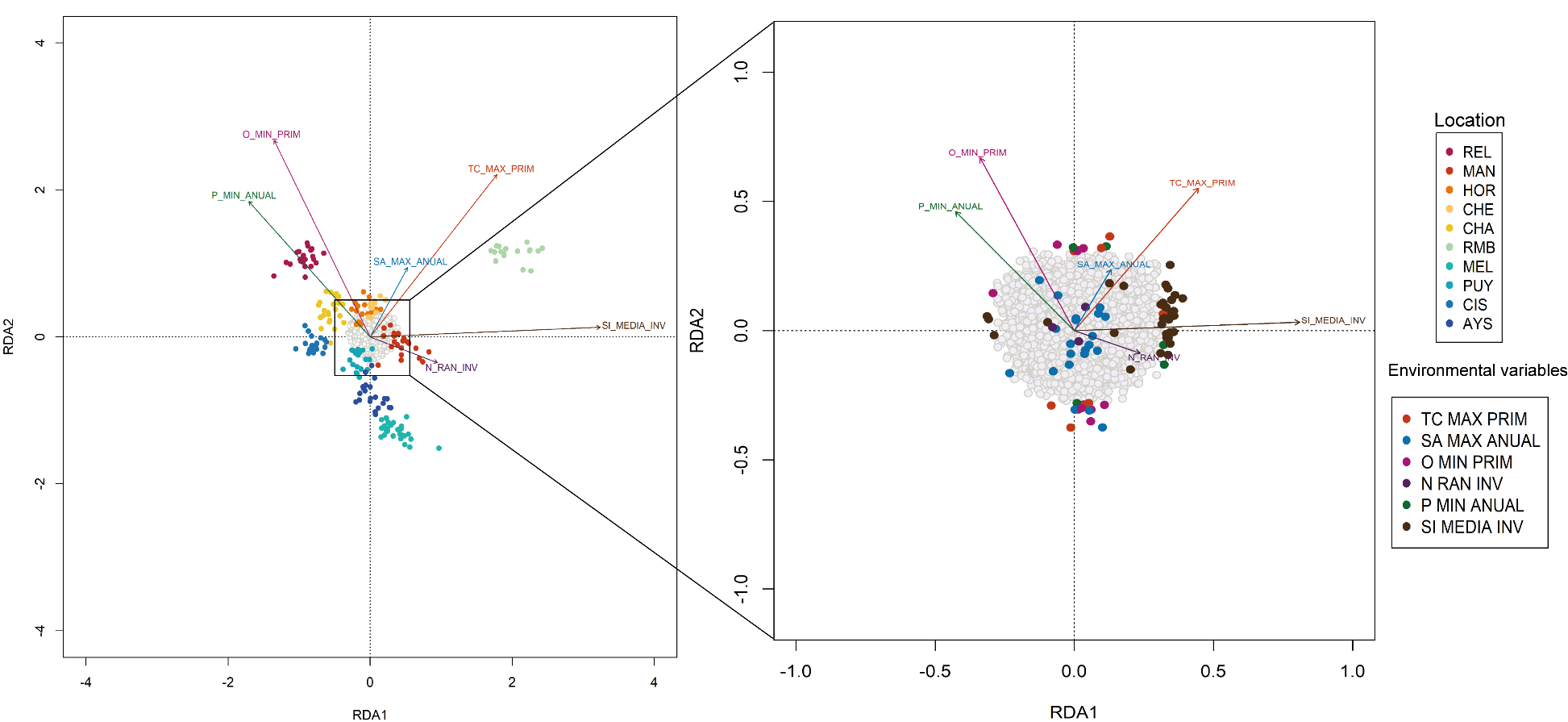
**

**Figure S7** Redundancy analysis (*RDA*) using the 11,961 loci. Arrows represent environmental variables (TC_MAX_PRIM: Spring maximum temperature, SA_MAX_ANUAL: Annual maximum salinity, O_MIN_PRIM: Spring minimum oxygen, P_MIN_ANUAL: Annual minimum phosphate, N_RAN_INV: winter nitrate range and SI_MEDIA_INV: Winter average silicate). A) Plots show the distribution of loci (gray dots) and genotypes of individuals (colored circles) are plotted according to their sampling location. B) Putative adaptative loci (colored circles) are plotted according to their predictor (environmental variables).

**
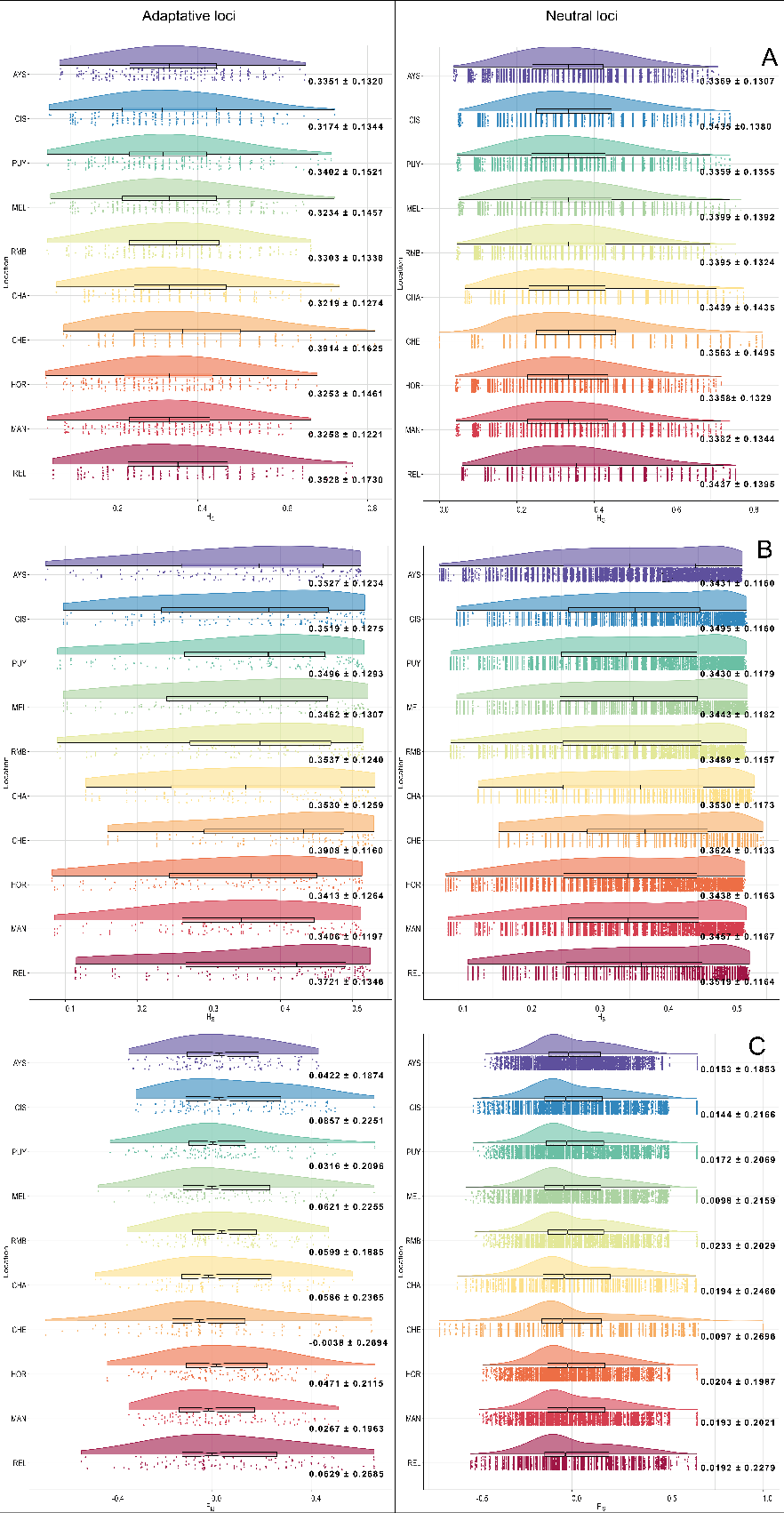
**

**Figure S8** Raincloud plots: Distribution of estimates of genetic diversity. A) H_O_ = observed heterozygosity, B) H_E_ = expected heterozygosity and C) F_IS_ inbreeding coefficient. Index was calculated using 131 loci for adaptive dataset and 9,536 loci for neutral dataset.


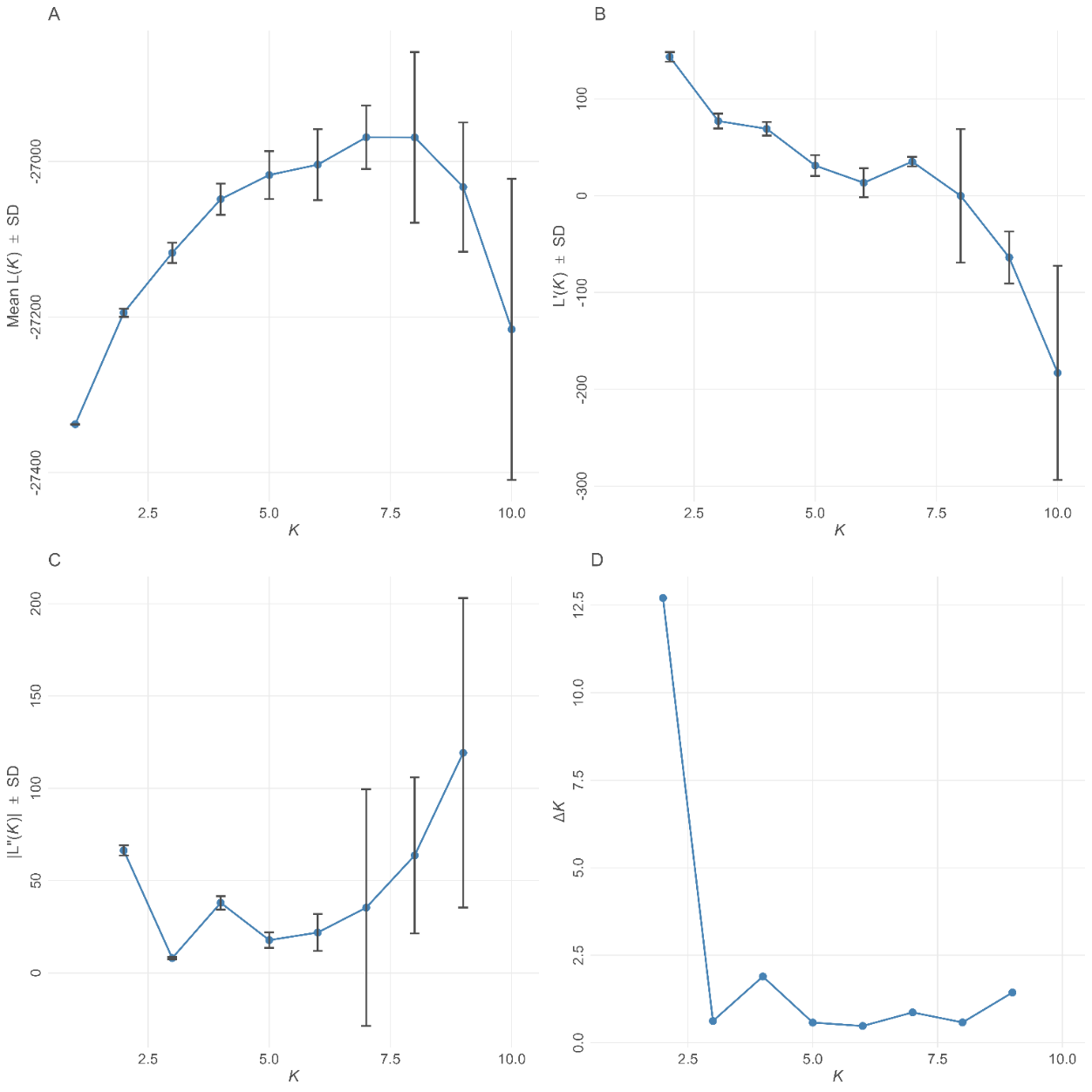


**Figure S9** Estimates of the number of clusters (K) for adaptive loci.Those using different statistics using the Evanno method to determine the ideal number of clusters by putatively adaptative loci. Error bars are standard deviation. A) Estimated log probability of the data for the 10 Structure runs at each K, B) first derivative, C) second derivative and D) ΔK, the rate of change in the log probability of data between successive K values.


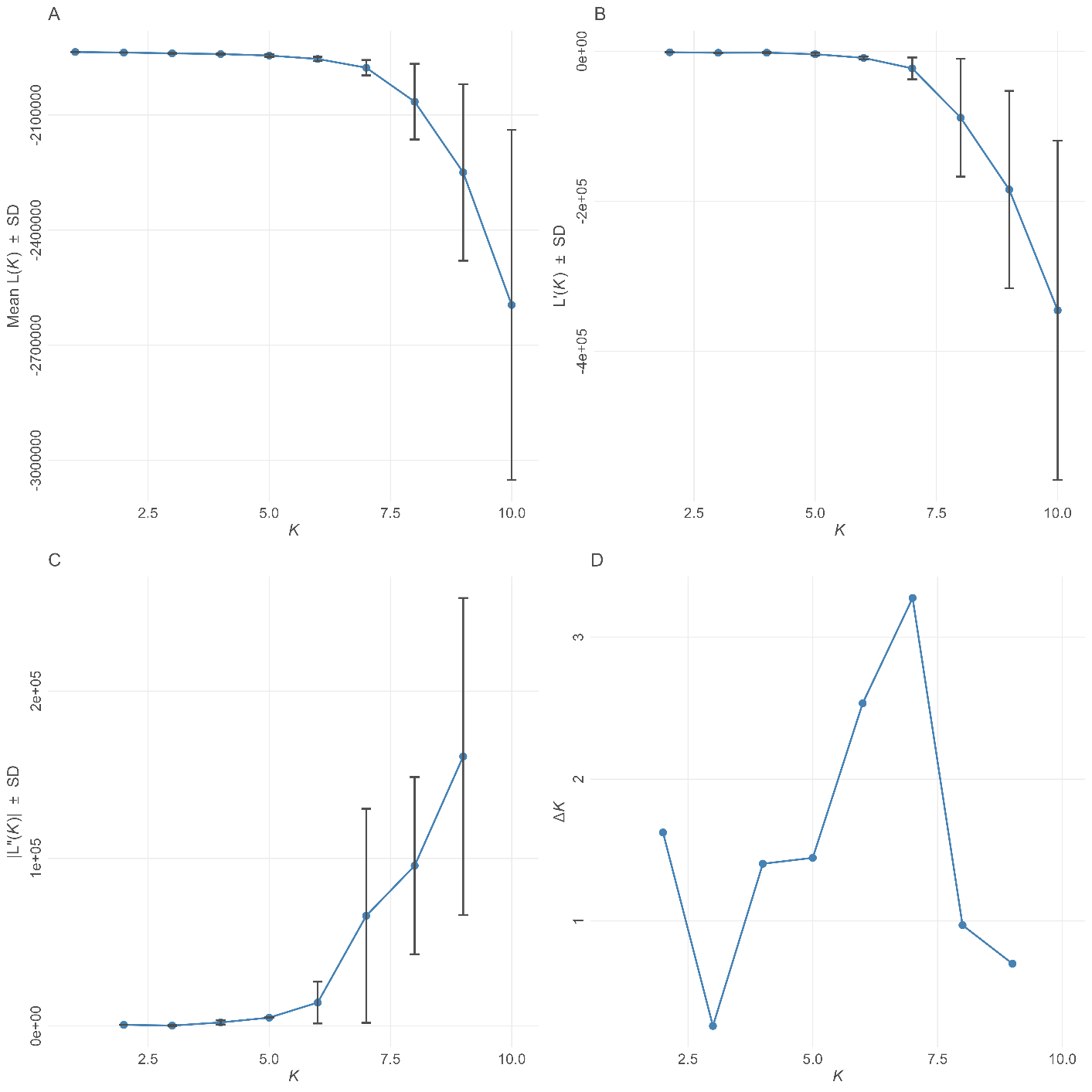


**Figure S10** Estimates of the number of clusters (K) for neutral loci. Using different statistics using the Evanno’s method to determine the ideal number of clusters by putatively neutral loci. Error bars are standard deviation. A) Estimated log probability of the data for the 10 Structure runs at each K, B) first derivative, C) second derivative and D) ΔK, the rate of change in the log probability of data between successive K values.


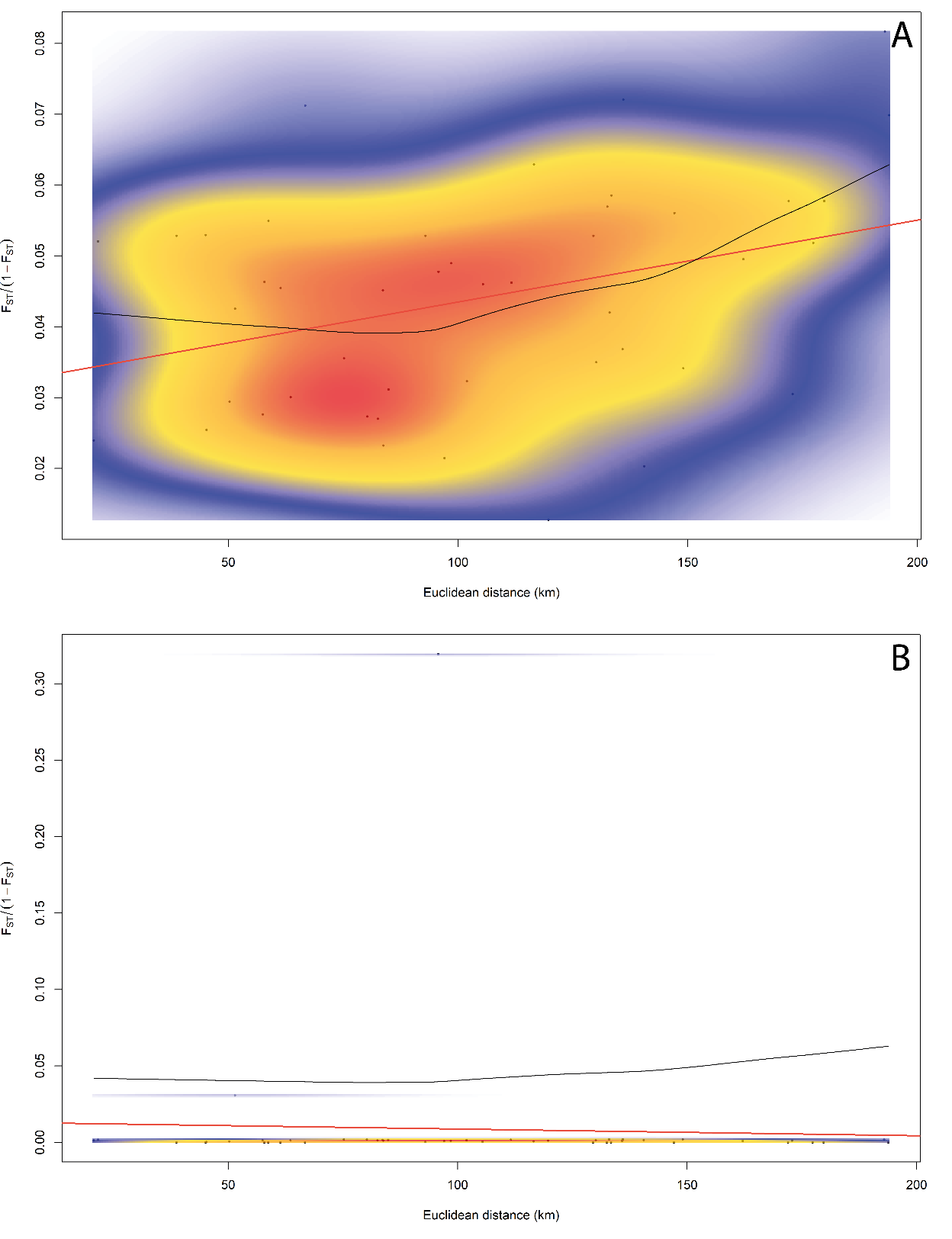


**Figure S11** Isolation-by-distance IBD among locations. A) 131 adaptative loci and B) 9,536 neutral loci. Local density of points plotted using two-dimensional kernel density estimation. The black line is the trend estimated by Loess regression red line indicates the linear regression. Color gradient represents density of observations (low = blue, red = high).


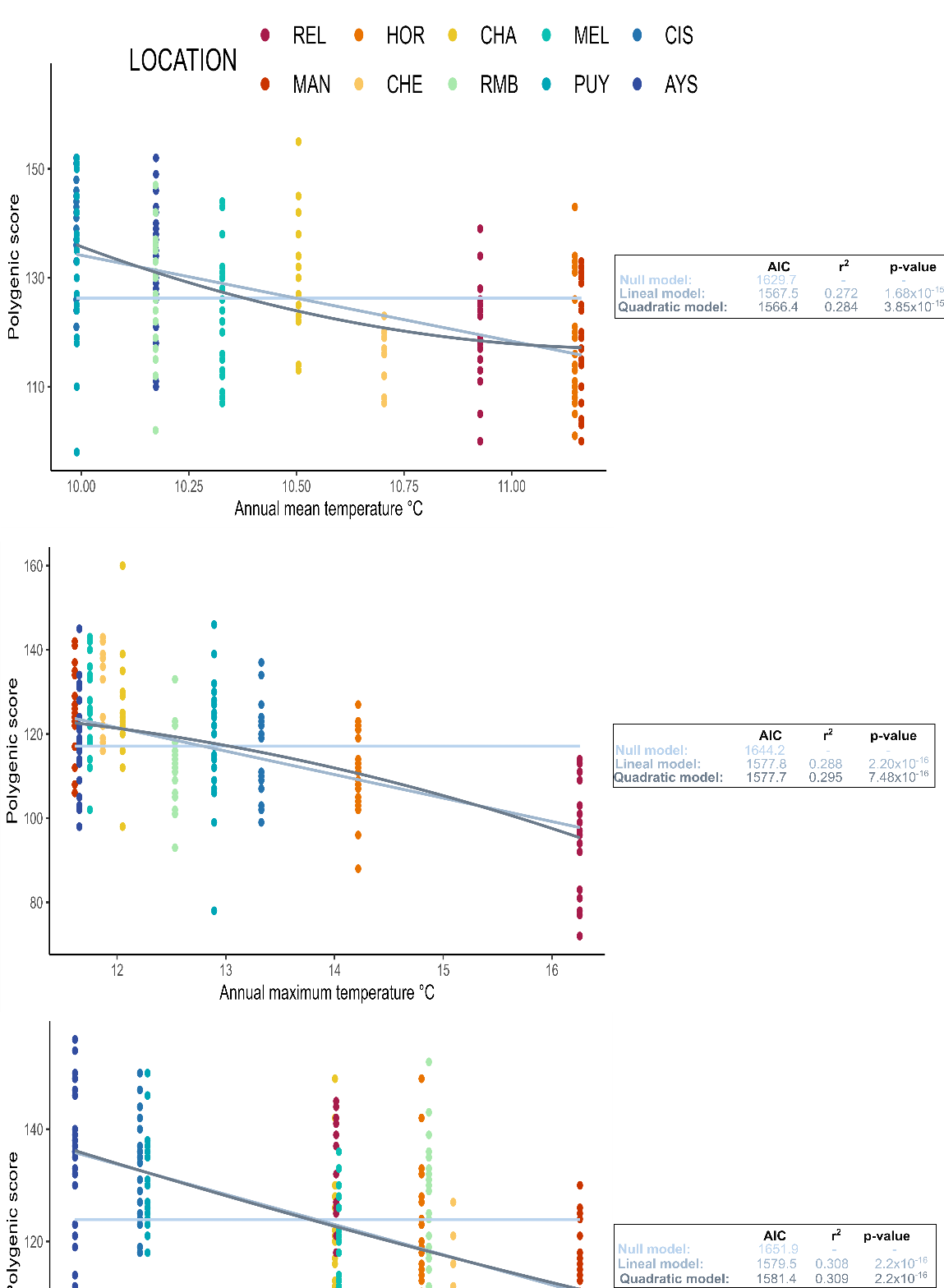


**Figure S12**  Correlations between additive polygenic scores (APS) based on A) Annual mean temperature B) Annual maximum temperature and C) Annual minimum temperature and 131 putative adaptative loci. Correlation coefficient (R^2^) and p-values and AIC are presented for each variable.


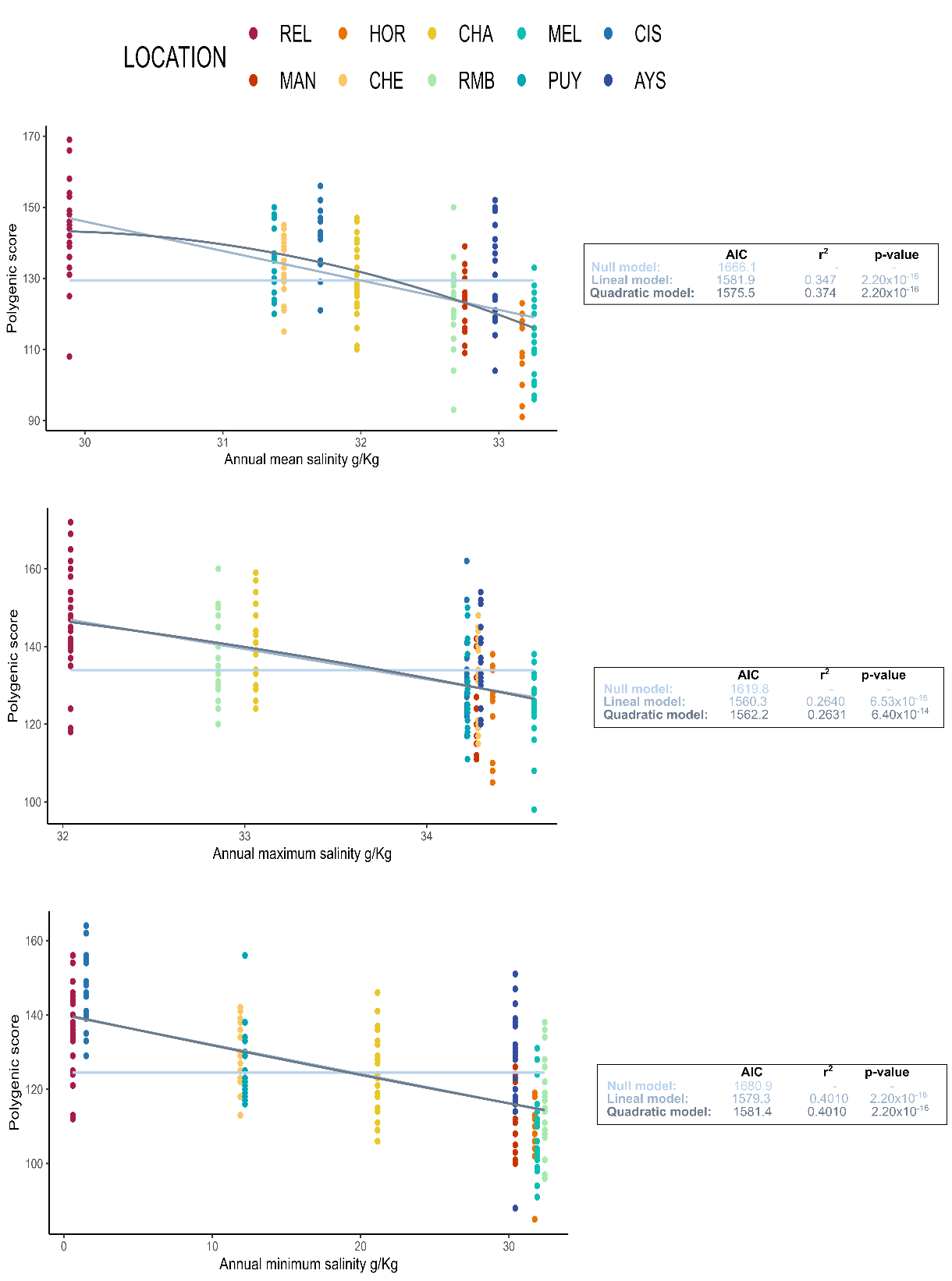


**Figure S13** Correlations between additive polygenic scores (APS) based on A) Annual mean salinity B) Annual maximum salinity and C) Annual minimum salinity and 131 putative adaptative loci. Correlation coefficient (R^2^) and p-values and AIC are presented for each variable.


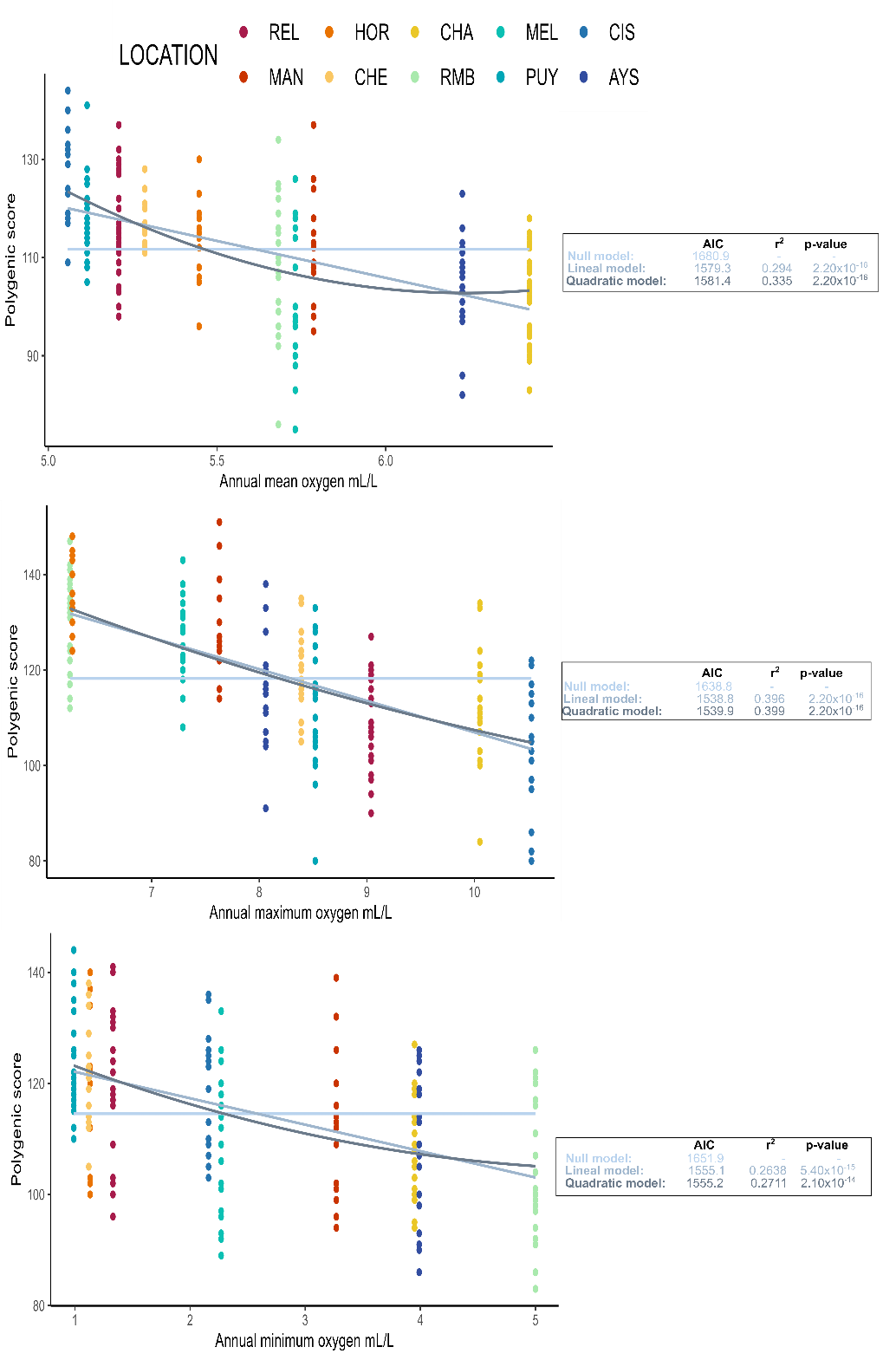


**Figure S14** Correlations between additive polygenic scores (APS) based on A) Annual mean oxygen concentration B) Annual maximum oxygen concentration and C) Annual minimum oxygen concentration and 131 putative adaptative loci. Correlation coefficient (R2) and p-values and AIC are presented for each variable.


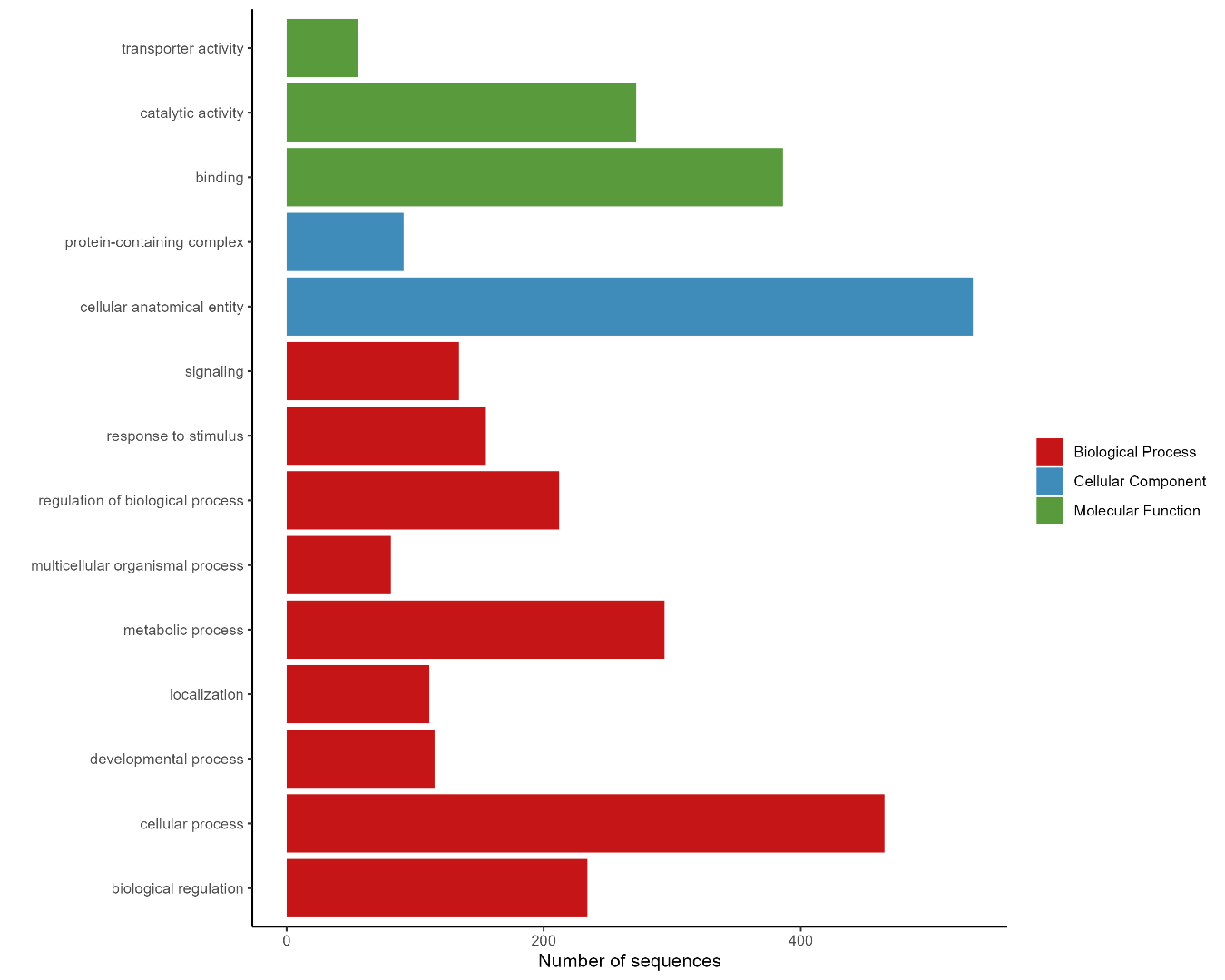


**Figure S15** Putative functional categorization and distribution according to Blast2GO.

**Supplementary Tables**

**Table S1**. Summary statistics for environmental variables are based on data collected from CIMAR-FIORDOS 1995-2018 at depths between 0 and 100 m.

* it was supplemented with data provided by IFOP (https://www.ifop.cl/chonos/) and Bio-ORACLE (<https://www.bio-oracle.org>)

**Table S2.** Summary biological information for individual of E. maclovinus for each sampling site used in this study.

| Location ID | Sample size | Total length of individuals (cm) | | | Individuals sex | | |
| --- | --- | --- | --- | --- | --- | --- | --- |
|  |  | Mean | Min. | Max. | Females | Males | Undetermined |
| PET | 23 | 30.42 | 16.60 | 49.10 | 11 | 3 | 9 |
| ANC | 25 | 8.46 | 4.40 | 11.00 | 0 | 0 | 25 |
| HOR | 28 | 15.14 | 9.00 | 30.00 | 0 | 0 | 28 |
| CHE | 18 | 22.58 | 19.00 | 26.50 | 0 | 0 | 18 |
| CHA | 18 | 36.88 | 31.80 | 43.00 | 12 | 5 | 1 |
| RMB | 26 | 43.61 | 37.50 | 54.00 | 23 | 2 | 1 |
| MEL | 27 | 35.76 | 31.00 | 42.00 | 22 | 3 | 2 |
| PUY | 26 | 10.84 | 6.00 | 26.00 | 0 | 0 | 26 |
| CIS | 24 | 23.44 | 6.50 | 38.00 | 6 | 1 | 17 |
| AYS | 31 | 25.29 | 16.90 | 48.00 | 5 | 4 | 22 |
| **Total** | **246** | **24.93** | **4.40** | **54.00** | **79** | **18** | **149** |

**Supplementary Tables**

**Table S3.** Description and parameters used for software with the PGD approach.

| **Software** | **Description** | **Parameters** | **References** |
| --- | --- | --- | --- |
| 1. *fsthet* | *fsthet* identifies loci with extreme F_ST_ values by calculating smoothed quantiles for the F_ST_-heterozygosity distribution, using its own empirical distribution instead of a null model due to changes in population structure. | We used a confidence level of 95% to detect *F*_ST_ outliers | (Flanagan & Jones 2017) |
| 1. *BayeScan* | *BayeScan* estimates the probability of selection for each locus using a Bayesian test that distinguishes between locus-specific selection effects (α) and population-specific demography effects (β), by measuring the discordance between global and population-specific allele frequencies. | We ran it with default parameters: 20 pilot runs of 5,000 iterations followed by 50,000 iterations with an additional burn-in of 50,000 and prior odd of 10. | (Foll & Gaggiotti 2008) |
| 1. *OutFLANK* | OutFLANK calculates a neutral distribution of F_ST_ values and assigns q-values to each locus to detect adaptive loci influenced by selection, without assuming a specific population's demographic history, reducing false positives. | We ran it with default parameters: LeftTrimFraction = 0.05, RightTrimFraction = 0.05, Hmin = 0.1. | (Whitlock & Lotterhos 2015) |
| 1. *PCAdapt* | *PCAdapt* performs a Principal Component Analysis (PCA) and computes the p-values for each locus to detect putative adaptative loci. | The most appropriate number of clusters K was chosen by testing K=1:10. We detected outliers using K=2 and p-values < 0.05 associated with Mahalanobis distances calculated with the q-value package. | (Dabney *et al.* 2010; Luu *et al.* 2017) |
| 1. *Arlequin* | *Arlequin* calculates observed heterozygosity (H_O_) to create a null distribution of F_ST_ values and associated p-values for each locus. | This was executed under a hierarchical island model with 20,000 simulations and 100 demes, which implements FDIST2 methodology. | (Excoffier & Lischer 2010) |

**Table S4.** Description and parameters used for software with the GEA approach.

| **Software** | **Description** | **Parameters** | **References** |
| --- | --- | --- | --- |
| 1. Latent factors mixed model (*LFMM*) | *LFMM* is a hierarchical Bayesian mixed model, which uses latent factors to correct population structure while fitting a linear regression between allele frequencies and environmental variables. | We estimated the number of ancestral populations K before the analysis testing from K 1 to 10 using the LEA v.3.2.0 R package and determined K from the cross-entropy criteria and Cattell's rule from the sNMF output. LFMM analyses were conducted separately for each environmental variable, with 10,000 iterations, and burning of 5,000 and five replicates. | (Cattell 1966; Devlin & Roeder 1999; Frichot *et al.* 2013, 2014; Frichot & François 2015) |
| 1. *BayeScEnv* | *BayeScEnv* detects putative adaptive loci by identifying loci that show large positive F_ST_ values (outside the neutral model F_ST_ distribution) that are significantly correlated with environmental variables. | We used 20 pilot runs, with 50,000 iterations each and an additional burn-in of 50,000 iterations for each of the six standardized environmental variables. | (de Villemereuil & Gaggiotti 2015). |
| 1. *Redundancy Analysis (RDA)* | *Redundancy Analysis (RDA)* is a multivariate ordination method to detect loci putatively under selection based on correlations with environmental variables. | We calculated the variance inflation factor (vif.cca function) to exclude variables with a value ≥ 5, and conducted linear regressions between allele frequencies and environmental variables at each locus. Using PCA, we produced ordination axes, identified putative adaptive loci (±3 SD from the mean), and calculated the Pearson correlation coefficient. | (Hair *et al.* 1995; Zuur *et al.* 2010; Rellstab *et al.* 2015; Forester *et al.* 2018) |
| 1. *Moran spectral outlier detection (MSOD)* | *MSOD* uses Moran eigenvector maps (MEM) to quantify the distribution of allele frequencies across a range of spatial scales represented by MEM spatial eigenvectors | We compared the power spectrum of each locus to the average power spectrum to identify outliers using z-scores and a cut-off of 0.05. Subsequently, we applied Moran spectral randomization (MSR) with 999 permutations using adespatial. | (Dray *et al.* 2006, 2021; Wagner & Dray 2015; Wagner *et al.* 2017) |
| 1. *Samβada* | *Samβada* is a spatial approach that uses multiples univariate logistic regression models to identify locus-environment associations and at the same time measures spatial autocorrelation | We coded individuals for the presence or absence of each SNP allele and measured the association with environmental parameters across sites using multiple logistic regression. P-values were calculated via a Wald Score Test and compared to a χ² distribution with one degree of freedom. | (Wald 1943; Stucki *et al.* 2017; Duruz *et al.* 2019) |

**Table S5.** Characterization of high-quality BLASTx. Matches obtained in comparison of *E*. *maclovinus* SNP against NCBI database. We only retained SNPs located in genes with putative functions that are compatible with the hypothesis of local adaptation.

| SNP | Detection method | Gene | Species | Protein name | e-value | Hit length | General function |
| --- | --- | --- | --- | --- | --- | --- | --- |
| 44791_149 | fsthet | slc39a6 (210632) | *Parambassis ranga* | zinc transporter ZIP6 isoform X2 | 1,712E-29 | 739 | MF: metal ion transmembrane transporter activity |
| 6291_214 | LFMM | slc8a2a (8218) | *Gymnodraco acuticeps* | sodium/calcium exchanger 2a | 2,000E-20 | 914 | MF: calcium: sodium antiporter activity; calmodulin binding; metal ion binding  BP: cell communication |
| 42_59 | LFMM | vtg3 (8218) | *Gymnodraco acuticeps* | Phosvitin | 6,131E-21 | 1266 | MF: lipid transporter activity; nutrient reservoir activity |

MF: Molecular function; BP: Biological
